# The receptor-like kinase NIK1 targets FLS2/BAK1 immune complex and inversely modulates antiviral and antibacterial immunity

**DOI:** 10.1038/s41467-019-12847-6

**Published:** 2019-11-01

**Authors:** Bo Li, Marco Aurélio Ferreira, Mengling Huang, Luiz Fernando Camargos, Xiao Yu, Ruan M. Teixeira, Paola A. Carpinetti, Giselle C. Mendes, Bianca C. Gouveia-Mageste, Chenglong Liu, Claudia S. L. Pontes, Otávio J. B. Brustolini, Laura G. C. Martins, Bruno P. Melo, Christiane E. M. Duarte, Libo Shan, Ping He, Elizabeth P. B. Fontes

**Affiliations:** 1 State Key Laboratory of Agricultural Microbiology, Huazhong Agricultural University, Wuhan, Hubei 430070 China; 2 The Provincial Key Lab of Plant Pathology of Hubei Province, College of Plant Science and Technology, Huazhong Agricultural University, Wuhan, Hubei 430070 China; 3 National Institute of Science and Technology in Plant–Pest Interactions, Bioagro, Viçosa, MG 36570.900 Brazil; 4 Department of Biochemistry and Molecular Biology, Universidade Federal de Viçosa, Viçosa, MG 36570.900 Brazil; 5 Department of Plant Pathology and Microbiology, Institute for Plant Genomics and Biotechnology, Texas A&M University, College Station, TX 77843 USA; 6 Department of Biochemistry and Biophysics, Institute for Plant Genomics and Biotechnology, Texas A&M University, College Station, TX 77843 USA; 7 Present Address: Federal Institute of Education from Goias, Science and Technology, Urutaí, GO 75790-000 Brazil; 8 Present Address: Instituto Federal de Educação, Ciência e Tecnologia Catarinense, Rio do Sul, SC 89163-356 Brazil; 9 Present Address: Laboratório Nacional de Computação Cientifica (LNCC), Petrópolis, RJ Brazil

**Keywords:** Virology, Plant immunity, Plant molecular biology

## Abstract

Plants deploy various immune receptors to recognize pathogens and defend themselves. Crosstalk may happen among receptor-mediated signal transduction pathways in the same host during simultaneous infection of different pathogens. However, the related function of the receptor-like kinases (RLKs) in thwarting different pathogens remains elusive. Here, we report that NIK1, which positively regulates plant antiviral immunity, acts as an important negative regulator of antibacterial immunity. *nik1* plants exhibit dwarfed morphology, enhanced disease resistance to bacteria and increased PAMP-triggered immunity (PTI) responses, which are restored by NIK1 reintroduction. Additionally, NIK1 negatively regulates the formation of the FLS2/BAK1 complex. The interaction between NIK1 and FLS2/BAK1 is enhanced upon flg22 perception, revealing a novel PTI regulatory mechanism by an RLK. Furthermore, flg22 perception induces NIK1 and RPL10A phosphorylation in vivo, activating antiviral signalling. The NIK1-mediated inverse modulation of antiviral and antibacterial immunity may allow bacteria and viruses to activate host immune responses against each other.

## Abstract (web summary)

Plants deploy numerous receptor-like kinases (RLKs) to respond to pathogens. Here the authors show that NIK1, an RLK that positively regulates antiviral immunity, negatively regulates the response to bacteria by modulating FLS2/BAK1 complex formation, suggesting crosstalk between bacterial and viral immunity.

## Introduction

Plants recognize potential pathogens mainly through two classes of distinct immune receptors^1–3^. The first class of immune receptors is cell-surface-associated pattern recognition receptors (PRRs), which are often represented by receptor-like kinases (RLKs) and receptor-like proteins (RLPs). PRRs recognize conserved structural motifs present in microbes, known as microbe- or pathogen-associated molecular patterns (MAMPs/PAMPs), or endogenous danger signals released by the host during wounding or pathogenic attack, termed as damage-associated molecular patterns (DAMPs)^4^. Perception of PAMPs by PRRs activates PAMP-triggered immunity (PTI), which inhibits a broad spectrum of potential pathogens, including bacteria, viruses, fungi, and oomycetes^1^. The second class of immune receptors includes intracellular immune receptors, which are designated as resistance (R) proteins^3,5^. These intracellular receptors directly or indirectly recognize avirulent effectors secreted by the pathogens into the host intracellular environment, thereby activating effector-triggered immunity (ETI), which is often associated with the hypersensitive response.

Plants counteract viral infection by employing both PTI and ETI^6–8^. An additional antiviral defence mechanism, which was uncovered recently, relies on host translation suppression mediated by a transmembrane immune receptor, nuclear shuttle protein (NSP)-interacting kinase 1 (NIK1)^9^. NIK1 belongs to the leucine-rich repeat (LRR) subfamily II of the RLK superfamily and was first identified as virulence targets of the begomovirus NSPs^10–13^. Loss of NIK1 function increases susceptibility to viral infection, whereas enhanced accumulation of NIK1 confers tolerance to begomovirus^10^. The mechanistic model for NIK1-mediated antiviral signalling stipulates that, upon virus perception, NIK1 undergoes dimerization to transphosphorylate a threonine position 474, leading to kinase activation^14,15^. Activated NIK1 phosphorylates the ribosomal protein RPL10, which in turn translocate into the nucleus, where it interacts with the RPL10-interacting Myb domain-containing protein (LIMYB) to repress the expression of translation-related genes^16–18^. The prolonged downregulation of translation machinery-related genes causes suppression of global protein synthesis, reducing the association not only of host messenger RNAs (mRNAs) but also of viral mRNAs with actively translating polysomes in infected cells, thereby preventing viral protein synthesis^18,19^. Therefore, this downregulation of cytosolic translation at least partially underlies the molecular mechanisms involved in NIK1-mediated antiviral defence.

NIK1 configuration resembles the structural organization of the somatic embryogenesis receptor kinases (SERKs), the other members of the LRRII-RLK subfamily^20^. This subfamily is further divided into (i) a cluster of five SERKs (1–5), (ii) a cluster of RLKs with unknown function and (iii) a cluster of NIK1-like receptors. Among the SERKs, SERK3, also named as brassinosteroid insensitive 1-associated kinase 1 (BAK1), is the most well-characterized subfamily member and, together with SERK4, functions as a co-receptor of several PRRs, such as flagellin sensing 2 (FLS2), elongation factor-thermo unstable receptor or plant elicitor peptide 1 receptor 1, which perceive specific PAMPs/DAMPs and trigger or amplify PTI^21–24^. In addition, members of SERKs complex with various endogenous peptide receptors to regulate plant development^25^.

Recently, we showed that loss of *NIK1* function up-regulates the expression of immune response-associated genes, suggesting a negative role of NIK1 in plant antibacterial immunity in contrast to its positive role in antiviral defence^9^. Emerging evidence has indicated that NIK1 exhibits a role in modulating PTI^26^. However, the underlying molecular link remains unclear. Here, we demonstrate that loss of *NIK1* function in *Arabidopsis* leads to increased resistance to bacterial pathogens. Furthermore, NIK1 associates with BAK1 and FLS2 and the NIK1 interaction was strengthened upon flagellin-derived flg22 treatment. Our results indicate that NIK1 inversely modulates antiviral and antibacterial immunity in plants, which may be dependent on the phosphorylation status of the protein.

## Results

### NIK1 function in resistance to viral and bacterial pathogens

Although NIK1 and BAK1 are conserved and belong to the LRRII-RLK subfamily, the mechanism of NIK1-mediated antiviral defence is distinct from that of BAK1-mediated PTI. Furthermore, the transcriptome induced by NIK1 activation seems to oppose the BAK1-mediated response^9,18^. To further examine the contribution of NIK1 to plant immunity, we analysed the differentially expressed genes in *nik1-1* seedlings (Supplementary Fig. 1a) (http://inctipp.bioagro.ufv.br/arabidopsisnik0/) using the eigenvector centrality method^27^ and the *Arabidopsis* pathogen interactome network database (http://interactome.dfci.harvard.edu/A_thaliana; Supplementary Fig. 2a). An important hub of upregulated genes in *nik1-1* is represented by genes involved in salicylic acid (SA) signalling. Upregulation of the SA signalling-associated *PR1* gene in *nik1-1* mutants, but not in *nik2-1* and *nik3-1* mutants, was confirmed by quantitative reverse transcription-PCR (qRT-PCR), which was associated with increased SA accumulation (Supplementary Fig. 2b, c). These results suggest that a subset of SA-related defence responses is constitutively activated in *nik1-1*-null mutant plants, consistent with the stunted growth, increased H_2_O_2_ accumulation and elevated cell death observed in *nik1-1* mutants (Supplementary Fig. 2d–f).

**Fig. 1:**
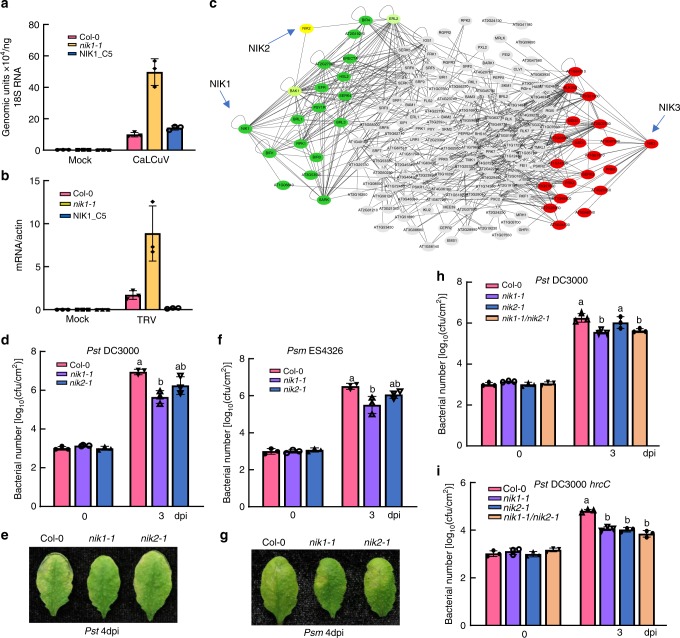
Opposite roles of NIK1 in resistance to viral and bacterial pathogens. **a** Quantification of CaLCuV genomic units in infected plants and mock-inoculated plants. Col-0, *nik1-1* and NIK1_C5 plants were inoculated with infectious CaLCuV DNA-A and DNA-B clones by biolistic delivery and viral DNA accumulation was determined by qPCR at 14 dpi using 18S rDNA as endogenous control. **b** Relative accumulation of TRV in infected and mock-inoculated plants. TRV from *N. benthamiana-*infected leaves was sap inoculated to *Arabidopsis* leaves and TRV expression was monitored by qRT-PCR. Error bars, 95% confidence intervals based on bootstrap resampling replicates of four independent (*n* = 4) experiment in **a**, **b**. **c** Major NIK1, NIK2 and NIK3 interacting clusters derived from the cell-surface interaction network of *Arabidopsis thaliana* leucine-rich repeat receptor-like kinase (CSILRR). The approximation maximum cut algorithm was used to rearrange the CSILRR network into clusters of NIK1, NIK2 and NIK3- directly interacting proteins. **d** Bacterial growth of *Pst* DC3000 post infection. Leaves of 4-week-old plants Col-0, *nik1-1* and *nik2-1* were hand infiltrated with bacterial suspensions of *Pst* DC3000 at a density of 5 × 10^5^ CFU/mL, and bacterial populations were quantified at 0 and 3 dpi. **e** Disease symptoms on leaves post *Pst* DC3000 infection. Images were taken at 4 dpi. **f** Bacterial growth of Psm ES4326 post infection. Similar experiments were performed as in **d** with Psm ES4326 inoculation. **g** Disease symptoms post Psm ES4326 infection. Images were taken at 4 dpi. **h** Growth of *Pst* DC3000 3 days post infection. Four-week-old plants of Col-0, *nik1-1*, *nik2-1* and *nik1-1*/*nik2-1* double mutants were hand-inoculated with *Pst* DC3000. **i** Growth of *Pst* DC3000 hrcC 3 days post infection. The above experiments were repeated three times with similar results. Different letters denote significant differences among bacterial number of distinct genotypes by the one-way ANOVA test (*p* < 0.05). Source data are provided as a Source Data file

Despite enhanced accumulation of free SA in *nik1-1* mutants, we have previously demonstrated that *nik1-1* displays an enhanced susceptibility phenotype to begomovirus *Cabbage leaf curl virus* (CaLCuV) infection (Fig. 1a)^10,16^. Elevated accumulation of viral DNA in *nik1-1* compared to Col-0 is most likely due to inactivation of the NIK1-mediated antiviral defence, which protects plant against DNA viruses^18,19^. However, the effectiveness of NIK1’s antiviral function against RNA viruses has not been evaluated. To address this issue, *Tobacco rattle virus* (TRV) was first propagated in *Nicotiana benthamiana* and rub-inoculated in *Arabidopsis*. The results revealed that relative levels of TRV genomic RNA were significantly higher in *nik1-1* than in Col-0 (Fig. 1b), indicating that increased endogenous SA and constitutive expression of SA-related genes are not sufficient to confer resistance to TRV. Enhanced susceptibility of *nik1-1* to TRV was due to the loss of *NIK1* function, as ectopic expression of *NIK1-GFP* restored the enhanced susceptibility phenotype in the knockout line (Supplementary Fig. 1d). These results indicate that NIK1 antiviral function is likely independent of the SA pathway and more important than over-accumulation of SA in controlling both DNA and RNA viral infections.

Constitutive activation of *nik1-1* defences is consistent with recent data from network-centric analyses of the LRR-based cell-surface interaction network (CSI^LRR^), which implicated NIK1 as one of the 35 predicted LRR-RLK pathogen effector targets and most influential spreaders of information in CSI^LRR^ network^26,28^. In this investigation, we applied the approximation maximum cut algorithm, which divided the CSI^LRR^ network into two major groups, clustering NIK1/NIK2 together and NIK3 as a separate group (Fig.1c). As this algorithm aims to identify a cut that maximizes connections between the two resulting sides, separation of a NIK1/NIK2 cluster from NIK3 might indicate that NIK1 and NIK3 differ in function and regulate at least two independent cellular processes. In contrast, NIK1 and NIK2 may share functional redundancies, although the degree of centrality demonstrates that NIK1 may strongly influence the spread of information^26^.

In contrast to viral infection phenotype, we found that *nik1-1*-knockout plants were more resistant to *Pseudomonas syringae* pv. *tomato* (*Pst*) DC3000 and *P. syringae* pv. *maculicola* (*Psm*) ES4326, as bacterial growth was significantly reduced in the *nik1-1*-knockout line relative to wild-type (WT) plants (Fig. 1d, f) and disease symptoms were attenuated in *nik1-1*-knockout mutants (Fig. 1e, g). NIK2 did not exhibit obviously negative modulation of resistance against *Pst* and *Psm*, suggesting differential relevance to the immune response between paralogous NIK1 and NIK2. The *nik1-1* allele has been previously described^10^ and further characterized herein using RNA-sequencing (RNA-seq) (Supplementary Fig. 1b, c). Importantly, we showed that inhibition of *Pst* growth in *nik1-1*-knockout mutants was restored by ectopic expression of *NIK1* (Supplementary Fig 3a, b). Therefore, loss of *NIK1* function enhances bacterial resistance, which is in marked contrast with the enhancement of viral susceptibility in *nik1-1*-null mutants.

To further characterize whether NIK1 and NIK2 exhibit redundant functionality in antibacterial immunity, we generated *nik1-1/nik2-1* double mutants (Supplementary Fig. 1e–h) and assayed for bacterial infection. Infection with *Pst* DC3000 was reduced in *nik1-1*, but not in *nik2-1*, while remaining inhibited in the *nik1-1/nik2-1* double mutant to the same extent as in *nik1-1*, strengthening the assumption that NIK2 does not affect resistance to *Pst* DC3000 (Fig. 1h). Likewise, we did not observe a synergistic or additive effect on the inhibition of *Pst* DC3000 *hrcC* growth, the nonpathogenic *Pst* DC3000 type III secretion mutant, in the double mutant *nik1-1/nik2-1* compared to bacterial growth in the single mutants (Fig. 1i). Notably, *nik2-1* mutants showed more susceptibility to the nonpathogenic strain *hrcC*, consistent with previous results^26^. While partial redundancy between these paralogous genes may exist with respect to modulating PTI, these results suggest that NIK1 and NIK2 may either modulate different, non-overlapping aspects of the same pathway or act upon distinct signal transduction pathways of the immune responses.

### Elevated early PTI responses in *nik1-1* mutants

**Fig. 2:**
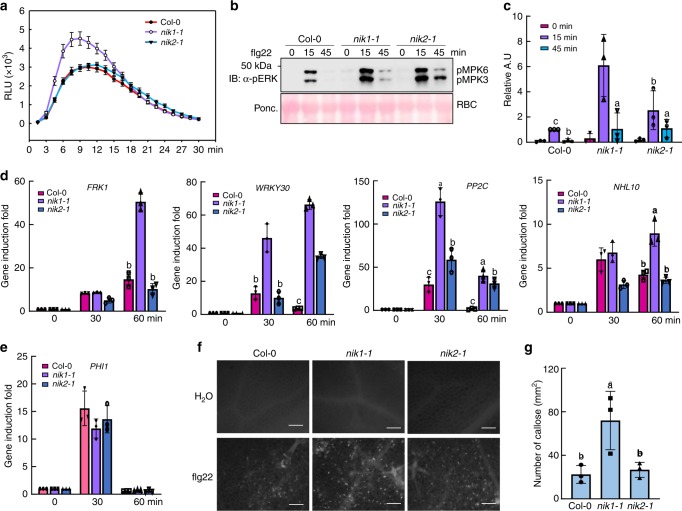
Elevated PTI early responses in *nik1-1-*null mutants. **a** Flg22-induced ROS burst in WT, *nik1-1* and *nik2-1* plants. Leaf discs from 5-week-old plants were treated with water or 500 nM flg22 for 30 min, and the relative light units (RLU) were detected. Values represent the mean ± SE (*n* > 10). **b** Flg22-induced MAPK activation in WT, *nik1-1* and *nik2-1* plants. Ten-day-old seedlings from the Col-0-, *nik1-1*- and *nik2-1-*knockout lines were treated with 100 nM flg22 and harvested at 0, 15 and 45 min post treatment. MAPK activation was monitored by immunoblotting total protein extracts with an α-pERK antibody (top panel). Protein loading was evaluated by Ponceau S staining for Rubisco (RBC) (bottom panel). **c** Quantitative data for MAPK activity signals. ImageJ was used to quantify signal intensity. Different letters denote significant differences by the one-way ANOVA test (*n* = 4, *p* < 0.05). **d** Relative induction of the PTI-associated marker genes *FRK1*, *WRKY30*, *PP2C* and *NHL10*. Ten-day-old seedlings were treated with 100 nM flg22 for 30 and 60 min. Total RNA was extracted for qRT-PCR analysis. Values represent the mean ± SD of five biological replicates. Different letters denote significant differences by the one-way ANOVA test (*p* < 0.05). **e** Gene induction of the CPK pathway-specific marker gene *PHI1* upon flg22 treatment. **f** Flg22-induced callose deposition in WT, *nik1-1* and *nik2-1* plants. Leaves of 5-week-old plants were infiltrated with 500 nM flg22 for 12 h and callose was detected by aniline blue staining. **g** Callose deposition triggered by flg22 treatment. The number in each image was quantified using the ImageJ software, and values represent the mean ± SD (*n* = 10). Different letters denote significant differences by the one-way ANOVA test (*p* < 0.05). Source data are provided as a Source Data file

To further examine the mechanism by which NIK1 negatively modulates antibacterial immunity, we activated PTI using flg22 and monitored the readouts of PTI activation in the *nik1-1*- and *nik2-1*-knockout mutants. As early responses, flg22 induced increased ROS accumulation and enhanced MAP kinase activation in the *nik1-1* line compared to Col-0, whereas the mitogen-activated protein kinase (MAPK) activity, but not reactive oxygen species (ROS) burst, was also elevated in *nik2-1*, although to a lesser extent (Fig. 2a–c). Stronger activation of MAPKs in *nik1-1* was followed by greater induction of the PTI-associated marker genes *FRK1*, *WRKY30*, and *PP2C*, whereas *WRKY30* and *PP2C were* also increased in the *nik2-1* mutant (Fig. 2d). However, the expression of calcium-dependent protein kinase pathway-specific marker gene *PHI1* was not further increased in *nik1-1* and *nik2-1* mutants (Fig. 2e), potentially indicating that NIK1 and NIK2 are not implicated in the Ca^2+^ signalling branch. Subsequently, increased callose deposits were observed in *nik1-1*, but not in *nik2-1* (Fig. 2f, g). As *nik2-1* mutants display moderate enhancement of immunity, NIK2 may regulate only certain branches of the PTI response. The enhanced resistance phenotype and PTI responses of *nik1-1* mutants were due to inactivation of *NIK1* function, as ectopic expression of *NIK1* in the *nik1-1* background restored the WT phenotype, as determined by ROS production and MAPK activation (Supplementary Fig. 3c–e). Furthermore, another transfer DNA (T-DNA) insertion mutant *nik1-2* supported less bacteria growth and displayed higher ROS production and MAPK activity compared to Col-0 (Supplementary Fig. 3f–h). Enhanced PTI activation was easily discernible in *nik1* mutants, demonstrating a more accentuated effect of NIK1 in the negative regulation of PTI among members of the NIK-like subfamily of LRRII-RLK proteins. Therefore, *NIK1* loss-of-function induces an enhanced flg22-induced immune response, a phenotype restored by *NIK1* complementation.

Interestingly, the *nik1-1-*knockout line accumulated higher levels of SA in a primed state, as infection with *Pst* caused the further accumulation of SA to a much higher levels than that observed in WT lines (Supplementary Fig. 4a). Therefore, the loss of *NIK1* function induces constitutive activation of immune responses, which are further amplified by the bacterial infection. SA signalling has previously been demonstrated to be required for early as well as late flg22 responses^29,30^. To understand whether the robustness of NIK1 modulation of PTI displayed in the *nik1-1* mutant is a result of increased SA accumulation in *nik1-1*, we generated *nik1-1/sid2-5* double mutants (Supplementary Fig. 4b, c). ROS burst in double mutants remained significantly higher than Col-0, but flg22-induced MAPK activity was greatly attenuated in *sid2-5* and *nik1-1/sid2-5* double mutants compared to *nik1-1* mutants (Supplementary Fig. 4d, e). Treatment with BTH, an agonist of the SA pathway, induces the accumulation of MPK3 and MPK6 in *Arabidopsis*;^31^ however, MPK3 and MPK6 were not more abundant in *nik1-1* mutants than Col-0, as monitored by α-MPK3 and α-MPK6 antibodies (Supplementary Fig. 4f, g). This result indicates that the stronger MAPK activity in response to flg22 is not due to increases in endogenous MPK protein levels.

As NIK1 negatively regulates most of the flg22-induced PTI responses, we next examined whether NIK1 requires the co-receptor BAK1 for the negative modulation of PTI. We generated *nik1-1/bak1-4* double mutants by crossing (Supplementary Fig. 5a), and disruption of *NIK1* and *BAK1* expression was confirmed by RT-PCR (Supplementary Fig. 5b). Upon flg22 treatment in the *nik1-1/bak1-4* double mutant, MAPK activation (Supplementary Fig. 5c, d) and induction of the defence genes, *PP2C* and *WRKY29*, were as low as in the *bak1-4* mutant (Supplementary Fig. 5e, f), indicating that NIK1 requires BAK1 to suppress flg22-triggered PTI responses. Collectively, these results indicate that NIK1 functions as a negative regulator of flg22-triggered immune responses.

### NIK1 binding to FLS2/BAK1 is enhanced by flg22 signalling

**Fig. 3:**
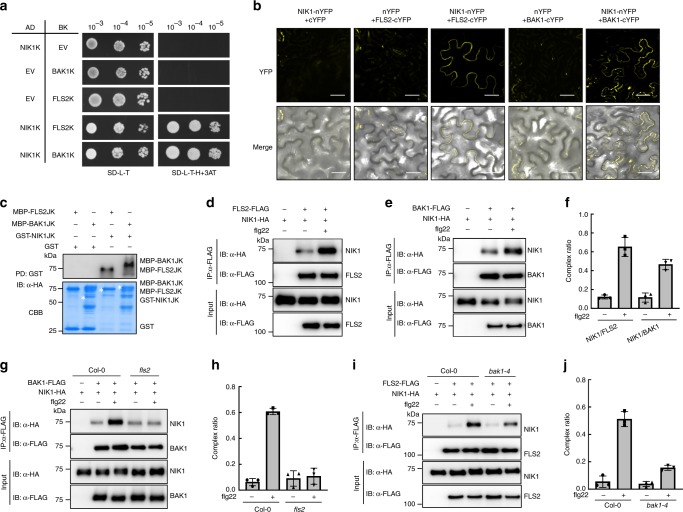
Interaction between NIK1 and the FLS2/BAK1 receptor complex is enhanced in response to flg22 signalling. **a** Interaction of NIK1 with BAK1 or FLS2 in Y2H assay. The kinase domains of NIK1, BAK1 or FLS2 were expressed in yeast as GAL4 activation domain (AD) fusions or binding domain (BD) fusions. EV indicates the empty vectors for either pGADT7 or pGBKT7. **b** In vivo interaction between NIK1 and BAK1 or FLS2 by BiFC analysis. Fluorescence (YFP) and bright field confocal images were acquired of tobacco leaves co-expressing the indicated fusion proteins in the presence of HC-Pro suppressor 48 h after agro-infiltration with the indicated DNA constructs. Scale bars = 20 µm. **c** NIK1 directly interacts with BAK1 or FLS2 in vitro. GST or GST-NIK1JK immobilized on glutathione Sepharose beads was incubated with MBP, MBP-FLS2JK or MBP-BAK1JK proteins. Beads were washed and pelleted for immunoblot analysis with α-HA antibody. PD, pull-down. **d**, **e** NIK1 associates with FLS2 or BAK1 and these interactions are strengthened by flg22 treatment. *Arabidopsis* protoplasts were co-transfected with NIK1-HA and FLS2-FLAG, BAK1-FLAG or an empty vector control. Protoplasts were treated with (+) or without (−) 100 nM flg22 for 15 min before harvesting. Co-IP was performed with α-FLAG Agarose (IP: α-FLAG), and proteins were analysed using immunoblots with an α-HA antibody (IB: α-HA). **f** Quantitative data for **d,** **e**. Signal intensity was quantified using the ImageJ software, and values represent the mean ± SD (*n* = 3). **g** Flg22-enhanced NIK1 and BAK1 interaction was abolished in a *fls2* mutant background. NIK1-HA and BAK1-FLAG were co-expressed in Col-0 or *fls2* protoplasts, and flg22 treatments were performed before samples collection. **h** Quantitative data for **g**. Images were quantified using the ImageJ software, and values represent the mean ± SD (*n* = 3). **i** Flg22-enhanced NIK1 and FLS2 interaction is largely reduced in a *bak1-4* mutant background. NIK1-HA and FLS2-FLAG were co-expressed in Col-0 or *bak1-4* protoplasts, and treated with flg22 before sample collection. **j** Quantitative data for **i**. Co-IP signals were quantified using the ImageJ software, and values represent the mean ± SD (*n* = 3). Source data are provided as a Source Data file

For the biochemical mechanism of negative modulation, we examined whether NIK1 directly interacts with the flg22 receptor FLS2 and with the co-receptor BAK1 by Y2H assay. Yeast expressing NIK1 kinase domain (NIK1K) and FLS2 kinase domain (FLS2K) or BAK1 kinase domain (BAK1K) grew on the deficient media, indicating that NIK1 interacts with BAK1 and FLS2 in yeast (Fig. 3a). We also demonstrated the interaction between NIK1 and these receptors by bimolecular fluorescence complementation (BiFC) assay in tobacco, since the reconstructed yellow fluorescent protein (YFP) signal was observed in cells containing NIK1-nYFP and FLS2-cYFP, but not in the control cells (Fig. 3b). BiFC assays confirmed that the formation of NIK1/FLS2 or NIK1/BAK1 complexes occurred in the plasma membrane of tobacco epidermal cells independent of the orientation of the NIK1 fusions (N terminus or C terminus of YFP) (Supplementary Fig. 6). *Escherichia coli*-produced MPB-FLS2JK or MBP-BAK1JK, also tagged with HA, were pulled down by GST-NIK1JK, indicating direct interactions in vitro (Fig. 3c).

In addition, we confirmed the in vivo association between NIK1 and FLS2 or BAK1 in *Arabidopsis* using co-immunoprecipitation (Co-IP) assays. NIK1-HA was co-immunoprecipitated from extracts co-transfected with BAK1-FLAG and FLS2-FLAG using anti-FLAG agarose without flg22 treatment (Fig. 3d, e) demonstrating the association of NIK1-HA with BAK1-FLAG and FLS2-FLAG in the absence of flg22. Interestingly, flg22 treatment promoted the NIK1 association with the FLS2/BAK1 complex, as demonstrated by the amount of co-immunoprecipitated NIK1 in the presence of flg22 (Fig. 3f). Therefore, binding of NIK1 to both FLS2 and BAK1 occurs constitutively and is further enhanced in response to flg22 treatment.

### NIK1 negatively modulates FLS2/BAK1 interaction and immunity

**Fig. 4:**
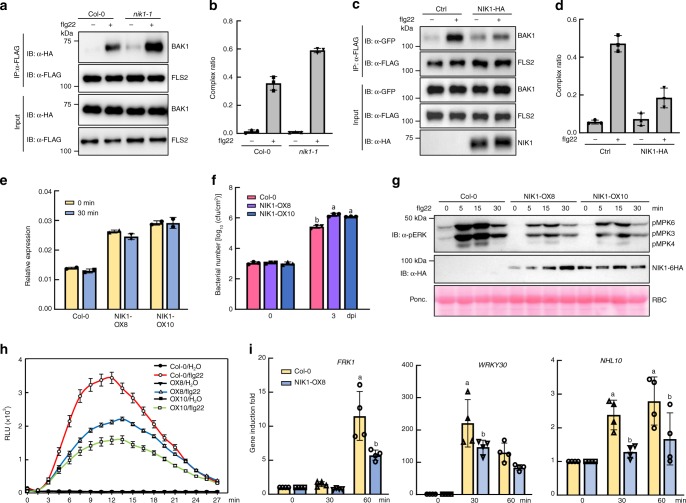
NIK1 negatively modulates the flg22-induced FLS2/BAK1 complex formation. **a** Flg22-induced FLS2 and BAK1 interaction is reinforced in *nik1-1* mutants. BAK1-HA and FLS2-FLAG were co-expressed in Col-0 or *nik1-1* mutant protoplasts, and samples were treated with flg22 for 10 min before Co-IP was performed. **b** Quantitative data for **a**. Co-IP images were quantified using the ImageJ software, and values represent the mean ± SD (*n* = 3). **c** Overexpression of *NIK1* impairs FLS2/BAK1 immune complex formation. BAK-GFP and FLS2-FLAG were co-expressed with or without NIK1-HA in Col-0 protoplasts; protoplasts were treated with flg22 before Co-IP was performed. Immunoprecipitation was carried out with an α-GFP antibody (IP: α-GFP), and the proteins were analysed using immunoblots with α-FLAG, α-GFP or α-HA antibodies. **d** Quantitative data for **c**. Co-IP images were quantified using the ImageJ software, and values represent the mean ± SD (*n* = 3). **e** *NIK1* expression level in WT and overexpression lines. Total RNA was isolated from Col-0 and overexpression lines and the expression of *NIK1* was monitored by qRT-PCR. **f** *Pst* DC3000 bacterial growth in WT and *NIK1*-OX lines. Four-week-old plants were hand-inoculated with pathogens, and bacterial populations were monitored at 0 and 3 dpi. Different letters denote significant differences among bacterial number of distinct genotypes by the one-way ANOVA test (*n* = 3, *p* < 0.05). **g** Flg22-induced MAPK activation in Col-0 and *NIK1*-OX lines. Ten-day-old seedlings from half MS plates were treated with flg22 and MAPK activation was monitored with an α-pERK antibody. **h** ROS burst is suppressed in *NIK1*-overexpressing transgenic lines. Leaf discs from soil grown plants were treated with water or 100 nM flg22 for 30 min, and RLU was measured. **i** qPCR analysis of PTI early response genes induction in *NIK1*-overexpressing plants. Ten-day-old seedlings were treated with flg22 for 30 and 60 min, and total RNA was extracted for qRT-PCR analysis. Values represent the mean ± SE of three biological replicates. Different letters denote significant differences by the one-way ANOVA test (*n* = 3, *p* < 0.05). Source data are provided as a Source Data file

The dynamics of NIK1 binding to the FLS2-BAK1 immune complex differ from that of another negative modulator of PTI, BIR2. Unlike NIK1, BIR2 does not interact with a cognate receptor and is released from BAK1 upon ligand perception^32^. These differences prompted us to investigate whether NIK1 directly modulates FLS2/BAK1 complex formation or inhibits signalling downstream of complex formation. We co-immunoprecipitated FLS2 with BAK1 from *nik1-1*-knockout lines or from *NIK1-*overexpressing protoplasts that had been treated with flg22 and compared the efficiency of complex formation in these samples to samples derived from Col-0. Loss of *NIK1* function increased the efficiency of flagellin-dependent FLS2/BAK1 complex formation (Fig. 4a, b). Further, we also showed that endogenous FLS2 and BAK1 do not over-accumulate in *nik1-1* mutants, indicating that the enhanced interaction was not due to elevated protein levels (Supplementary Fig. 4h, i). Consistently, NIK1-HA overexpression affected the flg22-induced FLS2/BAK1 complex formation, as the amount of FLS2 that co-immunoprecipitated with BAK1 in the *NIK1*-expressing sample was lower than that in the controls (Fig. 4c, d). These results indicate that NIK1 has a negative regulatory effect on BAK1/FLS2 complex formation, and this function is enhanced upon flg22 treatment to interfere with the activated receptor complex.

To further verify the negative role of NIK1 in the flg22-mediated PTI signalling pathway, we generated NIK1-6HA-overexpressing lines driven by the 35S promoter in the Col-0 WT background. Two independently transformed lines with similar protein expression levels were chosen for immune response assays (Fig. 4e). Overexpressing lines are more susceptible to *Pst* DC3000 infection compared to WT plants. The bacterial growth in overexpressing lines was approximately five-fold greater than that in WT (Fig. 4f). Furthermore, both flg22-induced MAPK activation and ROS burst were also largely supressed in *NIK1*-overexpressing lines compared to Col-0 (Fig. 4g, h). In addition, the flg22 induction of *FRK1*, *WRKY30* and *NHL10* was decreased in transgenic plants compared to WT (Fig. 4i). Collectively, these data demonstrate that innate immunity is compromised in *NIK1*-overexpressing plants, further substantiating the argument that NIK1 plays a negative role in FLS2 signalling.

### NIK1 interaction with FLS2 or BAK1 and phosphorylation

NIK1’s interaction with BAK1 and FLS2 is enhanced upon flg22 treatment (Fig. 3d, e). We hypothesized that flg22-induced stronger interactions between NIK1 and BAK1 or FLS2 were a result of NIK1 conformational changes resulting from flg22-induced formation of an active FLS2-BAK1 complex. This hypothesis accommodates the argument that NIK1 may be modified by phosphorylation that triggers for a conformational change that would favour interaction. To examine this hypothesis, we first monitored whether the flg22-induced higher affinity of NIK1 binding to BAK1 and FLS2 would require the presence of both receptor and co-receptor, a pre-requisite for complex formation. In this case, we monitored Co-IP of NIK1 with BAK1 in the *fls2* mutant and with FLS2 in the *bak1-4* mutant, comparing the resulting levels with those from Col-0 plants in the presence and absence of flg22. Under normal conditions, BAK1 and FLS2 are weakly bound to NIK1 in both the Col-0 and *fls2* or *bak1-4* lines (Fig. 3g, i). In Col-0, flg22 treatment increased the efficiency of interaction between NIK1 and BAK1. In contrast, flg22 did not increase NIK1 binding to BAK1 in *fls2* mutant lines (Fig. 3g, h) or to FLS2 in the *bak1-4*-knockout lines (Fig. 3i, j), suggesting that NIK1 may be a downstream target for FLS2-BAK1-mediated phosphorylation. This hypothesis was further examined using different approaches.

**Fig. 5:**
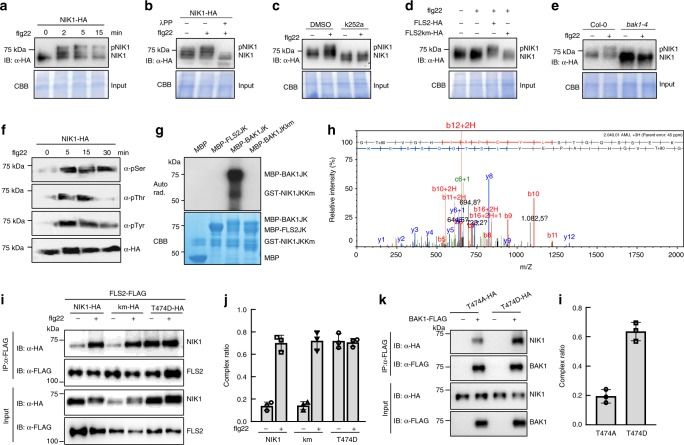
Flg22 induces BAK1-mediated NIK1 phosphorylation, which enhances NIK1’s affinity for receptors. **a** Flg22 perception triggers NIK1 rapid mobility shift. Protoplasts were transfected with NIK1-HA and treated with 100 nM flg22 for the indicated time points. Total input proteins were stained with Coomassie brilliant blue staining (CBB). **b** Verification of NIK1 in vivo phosphorylation by λPP treatment. Protein extracts from protoplasts transfected with NIK1-HA were treated with λPP following the standard protocol. **c** The kinase inhibitor K252a blocks flg22-induced NIK1 mobility shift. K252a was applied 1 h before flg22 treatment. Controls were solvent (DMSO) treatment. **d** Flg22-induced NIK1 phosphorylation requires FLS2 and its kinase activity. Protoplasts isolated from *fls2* mutants were transfected with NIK1-HA and empty vector control, FLAG-tagged FLS2 or FLS2 kinase mutant (FLS2km). **e** Flg22-mediated NIK1 phosphorylation requires BAK1. NIK1-HA was expressed in protoplasts of Col-0 or *bak1-4* mutants and flg22 was applied 10 min before samples collection. **f** Flg22-induced in vivo phosphorylation of NIK1 detected by different phospho-antibodies. Seedlings of NIK1-HA-overexpressing lines were treated with 100 nM flg22 for the indicated time points. NIK1-HA was immunoprecipitated from total protein extracts, fractionated by SDS-PAGE and immunoblotted with α-phosphoserine (α-pSer), α-phosphothreonine (α-Thr), α-phosphotyrosine (α-Tyr) and α-HA antibodies. **g** BAK1, but not FLS2, directly phosphorylates the NIK1 cytosolic domain. An in vitro kinase assay was performed using MBP-FLS2JK or MBP-BAK1JK as a kinase and GST-NIK1JKKm as the substrate. Phosphorylation was analysed by autoradiography (Upper), and the protein loading was shown by CBB (Lower). **h** NIK1 Thr474 is phosphorylated by BAK1 in vitro as shown by MS analysis. **i** Phosphorylation of NIK1 promotes its interaction with FLS2. FLS2-FLAG was co-expressed with NIK1-HA, NIK1Km-HA or NIK1-T474D-HA in protoplasts. Co-IP was performed with α-FLAG Agarose (IP: α-FLAG), and the proteins were immunoblotted with an α-HA antibody (IB: α-HA). **j** Quantitative data (*n* = 30 for **h**. **k** A NIK1 phosphomimetic form shows stronger interaction with BAK1. Co-IP assay was performed with the sample co-expressing BAK1-FLAG and T474A-HA or T474D-HA using α-FLAG Agarose. **l** Quantitative data from three biological replicates for **j**. Source data are provided as a Source Data file

We first observed that NIK1 shows a rapid mobility shift upon flg22 treatment, which was displayed by the presence of multiple bands on a 7.5% sodium dodecyl sulfate-polyacrylamide gel electrophoresis (SDS-PAGE) gel (Fig. 5a). This mobility shift was reversed by phosphatase (λPP) treatment, indicating that the band shift was due to NIK1 phosphorylation (Fig. 5b). In addition, pre-treatment with the kinase inhibitor K252a blocked the flg22-induced band shift (Fig. 5c). Importantly, the NIK1 band shift was not observed in *fls2* and *bak1-4* mutant backgrounds, but was restored by expression of FLS2, but not of a FLS2 kinase mutant (km), in *fls2* mutants (Fig. 5d, e). Flg22-induced phosphorylation of NIK1 was further demonstrated by probing immunoprecipitated NIK1-HA with anti-pThr, pSer and pTyr antibodies after flg22 treatment (Fig. 5f). Collectively, these results establish that flg22 induces phosphorylation of NIK1, which is dependent on the formation of an active BAK1-FLS2 complex.

To identify the kinase that is responsible for NIK1 phosphorylation, an in vitro kinase assay was performed using juxtamembrane domain and kinase domain of FLS2(FLS2JK) or BAK1JK as the kinase and NIK1JKkm as the substrate. The results demonstrated that BAK1JK directly phosphorylates NIK1JKkm (Fig. 5g). FLS2 is a non-RD kinase whose kinase activity is difficult to detect in vitro. As previously demonstrated, NIK1 undergoes dimerization and autophosphorylation on Thr474 to activate NIK1-mediated antiviral signalling^18^. We first showed that a peptide from the NIK1 activation loop is phosphorylated on T474 using purified recombined GST-NIK1JK protein (Supplementary Fig. 7a–c), as determined by mass spectrometry analysis (Supplementary Fig 7d, e). Likewise, the purified GST-BAK1JK fusion protein, but not a GST-FLS2JK fusion, phosphorylated the NIK1 A-loop peptide on the Thr474 residue, as determined by mass spectrometry (Fig. 5h, Supplementary Fig. 7f). These data suggest that BAK1 may directly phosphorylate NIK1 at Thr474 in the FLS2 signalling pathway.

The higher affinity of NIK1 for the FLS2/BAK1 immune receptor complex may be facilitated by phosphorylation on the Thr474 residue, as a phosphomimetic mutant NIK1-T474D displayed stronger association with FLS2 than NIK1 in the absence of flg22 stimulation (Fig. 5i, j). Similarly, increased levels of a phosphomimetic variant were co-immunoprecipitated by BAK1 compared to non-phospho variant (Fig. 5k, l). Interaction of NIK1-T474D with BAK1 and FLS2 was also monitored by BiFC (Supplementary Fig. 6). The reconstituted YFP signal mediated by T474D interactions with the receptors was consistently stronger than those mediated by NIK1 interactions.

We also generated a NIK1 kinase dead mutant by replacing the conserved Arg340 with Ala (hereafter NIK1^km^) in the ATP binding pocket, which is no longer capable of autophosphorylation (Supplementary Fig. 8a). Interestingly, NIK1^km^ displayed an unaltered flg22-triggered band shift (Supplementary Fig. 8b) and enhanced association with the FLS2/BAK1 immune complex similar to the WT form (Fig. 5i, Supplementary Fig. 8c). Furthermore, NIK1^km^ maintains suppression of FLS2/BAK1 receptor complex formation when co-expressed in *Arabidopsis* protoplasts (Supplementary Fig. 8d). Furthermore, MAPK activation and FRK1 reporter gene induction were suppressed in response to NIK1^km^ expression in *nik1-1-* null alleles, further indicating that the biological function of NIK1 in attenuating the FLS2 immune responses is not affected by its kinase activity (Supplementary Fig. 8e, f). Therefore, autophosphorylation of NIK1 is not required for its high affinity for the immune complex FLS2/BAK1 or its regulatory roles in FLS2 signalling. Collectively, these results indicate that NIK1 serves as a downstream target of the flg22-induced BAK1-FLS2 complex, which phosphorylates NIK1 to enhance NIK’s affinity for FLS2 and BAK1.

### Flg22-induced NIK1 phosphorylation elicits antiviral defence

**Fig. 6:**
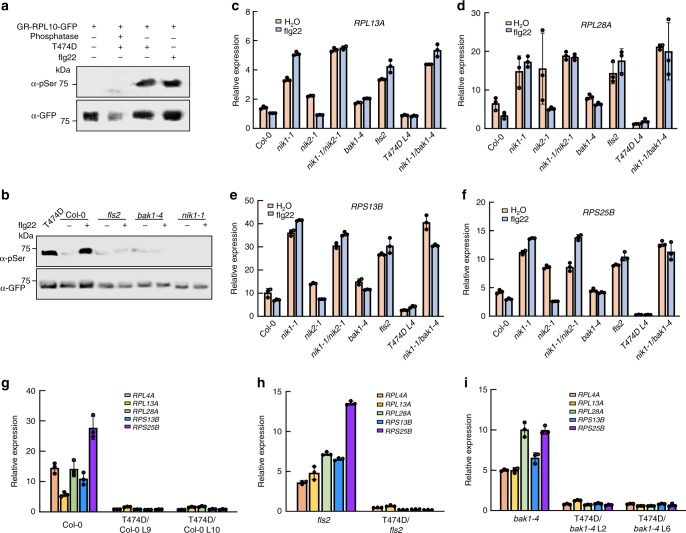
Flg22 induces RPL10 phosphorylation and the translation control branch of antiviral signalling in a NIK1-, FLS2/BAK1-dependent manner. **a** Flg22 treatment induces RPL10 phosphorylation. RPL10 phosphorylation was detected by immunoblotting with an α-phosphoserine (α-pSer) antibody (top) and RPL10 protein is shown using an α-GFP antibody. The experiment was repeated three times with identical results. **b** RPL10 phosphorylation requires the FLS2/BAK1 receptor complex. RPL10 was expressed in protoplasts isolated from Col-0, *fls2*, *nik1-1* or *bak1-4* mutants for 12 h, and then flg22 treatment was performed. T474D-mediated RPL10 phosphorylation was used as a positive control. The experiment was repeated twice with similar results. **c**–**f** Flg22-induced downregulation of *RP13A*, *RPL28A*, *RPS13B* and *RPS25B* is dependent on NIK1 and FLS2/BAK1. Seedlings of indicated plants were treated with flg22 for 3 h, and expression of ribosomal genes was analysed by qRT-PCR. Data are shown as the mean ± SE (*n* = 3). **g** Overexpression of NIK1-T474D in WT Col-0 suppresses expression of ribosomal genes. qRT-PCR analysis of RP expression levels in Col-0- and T474D-overexpressing lines. **h**, **i** FLS2 and BAK1 are not required for the suppression of ribosomal genes by NIK1-T474D overexpression. Ribosomal marker gene expression levels were detected in *fls2*, *bak1-4* mutants and NIK1-T474D-overexpressing lines. The respective 95% confidence interval limits were estimated based on bootstrap resampling replicates of three independent (*n* = 3) experiments and three technical repeats. Source data are provided as a Source Data file

We demonstrated that flg22 induces NIK1 Thr474 phosphorylation, which is mediated by an active immune complex, FLS2/BAK1. As Thr474 phosphorylation of NIK1 is critical for NIK1-mediated antiviral immunity, we then examined whether flg22-induced phosphorylated NIK1 activates the downstream translational control branch. Both flg22 treatment and NIK1-T474D expression induced RPL10 phosphorylation as shown by an anti-phosphoserine antibody that was eliminated by phosphatase treatment (Fig. 6a). T474D-induced phosphorylation of RPL10 was used as a positive control^16^. As expected, flg22 treatment did not trigger RPL10 phosphorylation in the *nik1-1* mutant, but it was also not triggered in *bak1-4*, *fls2* mutants or *nik1-1/bak1-4* double mutants, confirming that flg22-induced activation of NIK1 antiviral signalling depends on FLS2/BAK1-mediated phosphorylation of NIK1 (Fig. 6b). This interpretation was further substantiated by analysing flg22-mediated regulation of the downstream components of NIK1-mediated antiviral signalling.

Downregulation of ribosomal protein (RP) genes is the downstream output of NIK1/RPL10/LIMYB-mediated defence signalling;^18^ therefore, we speculated that flg22-induced NIK1 and RPL10 phosphorylation might also supress RP gene expression. Based on published flg22 transcriptional profiles^33^, we found RP genes were progressively downregulated by flg22 in a time-dependent manner post treatment (Supplementary Fig. 9a). To further confirm the flg22-mediated suppression of RP expression, we treated Col-0, *nik1-1*, *fls2* and *bak1-4* seedlings with flg22 and monitored expression of RP marker genes 30 min and 3 h post-treatment. While flg22 treatment for 30 min did not induce downregulation of RP genes (Supplementary Fig. 9b), in 3 h post-treatment, the expression of the RP marker genes *RPL13A*, *RPL28A*, *RPS13B*, *RPS25B* and *RPL4A* was suppressed in Col-0 plants (Fig. 6c–f and Supplementary Fig. 9c). However, flg22-mediated inhibition of marker genes was completely abolished in in *nik1-1* seedlings and *nik1-1/nik2-1* double mutants. Likewise, flg22 treatment did not repress expression of RP marker genes in *fls2* and *bak1-4* or *nik1-1/bak1-4* mutants, confirming that FLS2/BAK1 is required for NIK1 and RPL10 phosphorylation (Fig. 6c–f and Supplementary Fig. 9c). Flg22 did not promote further repression of RP gene expression in T474D-overexpressing lines, demonstrating that NIK1 phosphorylation is downstream of the flg22-induced FLS2/BAK1 complex formation. Consistent with this interpretation, ectopic expression of the NIK1-T474D mutant in two independently transformed *bak1-4* lines (Supplementary Fig. 9f) and one transformed *fls2* line (Supplementary Fig. 9e) suppressed expression of RP marker genes to the same extent as the T474D expression in stably transformed Col-0 lines (Fig. 6g–i, Supplementary Fig. 9d). Collectively, our results indicate that flg22 activates NIK1-mediated antiviral defence through BAK1-mediated phosphorylation of NIK1, establishing potential interplay between two innate immune pathways.

## Discussion

As the first line of innate immunity, PTI is rapidly activated upon pathogen perception primarily to defeat host non-adapted or non-host pathogens^4,34^. PRRs are ligand-dependent receptors with a high affinity for PAMPs; thus, receptor activation, which often includes association with a co-receptor, must be tightly controlled, because constitutive activation of defence responses adversely affects plant fitness and growth^35^. Thus, the immune system is negatively controlled by several layers of regulation, including ubiquitin-mediated degradation of immune receptor PRRs^36^, inhibition of active immune complex formation^32^, modulation of PRR phosphorylation^37^ and negative regulation of downstream components^38^. Here, we gained new insight into the mechanisms underlying the attenuation of PRR activation by preventing these receptors from constitutively signalling. We provided several lines of evidence that conclusively implicate the positive antiviral immune component NIK1 as a negative regulator of FLS2 and BAK1 receptor complex formation both before and after flg22 perception. First, we showed that loss of *NIK1* function constitutively activates SA-related plant defences, conveying additional resistance to bacterial pathogens. Second, *NIK1* inactivation enhanced PTI responses to flg22 treatment, as determined by increased ROS production, enhanced MAPK activation, induction of PTI-associated marker genes and increased callose deposition in *nik1-1* plants relative to WT plants. Third, under normal conditions, NIK1 interacts constitutively with FLS2 and BAK1, which seems to prevent activation of an autoimmune response. Finally, we showed that NIK1 controls FLS2/BAK1 heterodimerization, as the efficiency of complex formation and the immune responses depend on basal concentrations of NIK1. However, we also showed that NIK1 is not released from BAK1 and FLS2 upon flg22 treatment, instead it remained tightly associated with FLS2 and BAK1. This unprecedented mechanism of PTI inhibition contrasts sharply with previously described mechanisms of PTI inhibition by RLKs, such as BIR2^32^ and BIR3^39^, which interact with BAK1 under normal conditions but is released from BAK1 upon flg22 perception.

We also provided several lines of evidence indicating that the higher affinity of NIK1 for the FLS2/BAK1 complex may be a result of BAK1-mediated NIK1 phosphorylation. We first showed that flg22-induced higher affinity of NIK1 for the immune receptor or its co-receptor requires the presence of the other signalling partner, indicating the need for formation of an active immune complex. Next, we demonstrated that flg22 induces NIK1 phosphorylation in vivo, which requires BAK1 and FLS2, and BAK1 phosphorylates NIK1 on the Thr474 residue in vitro. Finally, we showed that the phosphomimetic NIK1 variant T474D interacts more strongly with BAK1 and FLS2 than does WT NIK1, whereas the NIK1 mutant NIK1km, which is no longer capable of autophosphorylation, retains the NIK1 standard binding profile. This latter result rules out the possibility that a higher NIK1 affinity for BAK1 and FLS2 is due to NIK1 autophosphorylation.

Furthermore, in this investigation, we showed that *nik1-1-* knockout mutants were more resistant to *Pst* and *Psm*, which differed from the previously reported phenotype displayed by the *nik1-2* alleles^26^. Under our experimental condition, the *nik1-2* mutant displayed modest enhanced resistance to *Pst* compared with *nik1-1*, which was further supported by stronger PTI responses compared with WT. Our results suggest that *nik1-2* mutant is a relatively weak allele compared with *nik1-1* mutant, which may be due to differences in T-DNA insertion within the *NIK1* locus (Supplementary Fig. 1a). Precedents in the literature have shown that homozygous T-DNA insertion lines within the same locus display phenotypic variation with respect to several traits^40^. In the case of the *nik1-1* alleles, we further demonstrated by complementation assays that the enhanced resistance phenotype to *Pst* was a result of *NIK1* disruption. We also showed that *nik1-1* alleles display enhanced SA content and develop cell death constitutively. Based on network-centric analyses of the leucine-rich repeat (LRR)-based cell-surface interaction (CSI^LRR^) network, NIK1 is predicted to function broadly in different layers of plant defences and to bind to BIR1 (Fig. 1c), a negative regulator of SOBIR1 and cell death^41^.

**Fig. 7:**
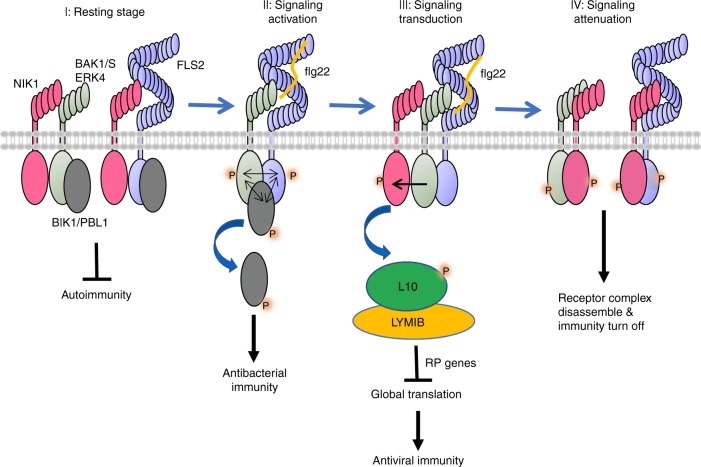
Working model for NIK1 roles in FLS2 signalling pathway and flg22-induced translational control of NIK1 signalling. (I) At the resting stage, NIK1 associates with FLS2 or BAK1 to prevent autoimmunity without pathogens invasion. (II) Upon bacterial infection, flg22 is perceived by the immune receptor FLS2, which induces the heterodimerization with BAK1, also including RLCK BIK1 and its homologues. Phosphorylation events initiates the immune signalling, and BIK1 is released from the membrane to activate downstream signal. (III) During signalling transduction, BAK1 may phosphorylate NIK1 on Thr474 residues. Phosphorylated NIK1 leads to activation of an antiviral signal through RPL10 phosphorylation and suppression of translational machinery-related gene expression. (IV) At signalling attenuation stage, phosphorylated NIK1 exhibits higher affinity to FLS2 and BAK1, and these interactions may lead to receptor complex instability or disassembly, followed by FLS2 ubiquitination and degradation

Our current findings, together with previous results^16,18^, suggest a mechanistic model for the interaction of the NIK1-mediated antiviral signalling pathway with the antibacterial immunity system (Fig. 7). In the absence of bacterial and viral infection, NIK1 is bound to FLS2 and BAK1 to prevent activation of an autoimmune response. NIK1 may control FLS2/BAK1 complex formation, as the efficiency of the immune response depends on basal concentrations of NIK1. Upon bacterial infection, flg22 is sensed by the FLS2 extracellular domain, which in turn recruits BAK1 into an active immune complex that phosphorylates NIK1 on its crucial Thr474 residue. This modification may result in two important effects in innate immunity. First, NIK1 more effectively binds to the FLS2/BAK1 receptor complex, thus inhibiting constant signalling activation and preventing immoderate immunity. Furthermore, the flg22-induced phosphorylation activates NIK1 to initiate the transduction of an antiviral signal through RPL10 phosphorylation and suppression of translational machinery-related gene expression. Therefore, flg22 treatment may promote plant resistance to viruses in a NIK1-dependent manner. As begomovirus infection has been previously demonstrated to suppress NIK1 antiviral function through the viral NSP suppressor, the sequential order of events may invoke a suppression-dependent mechanism to relieve NIK1-mediated negative modulation of the immune response. Therefore, viral infection prior to bacterial attack may prime the host for enhanced resistance against bacteria. The potential interaction and mechanism between these two innate immune pathways are worth additional study in the future.

A pitfall in these crosstalk studies, however, may be the finding that viral infection inhibits PTI directly via viral protein suppressors^39,42,43^. Compelling evidence has invoked the classic transmembrane PRR-mediated PTI as part of the plant defence arsenal against viruses^41,44,45^. Mutation of the PTI co-receptors BAK1 or BKK1 enhances susceptibility to RNA virus infection, demonstrating that they are required to build an effective defence against RNA viruses in *Arabidopsis*^41,46^. Likewise, *serk1*-knockout lines are more susceptible to virus infection^45^, and the *Arabidopsis* double mutant *bak1-5/bkk1* displays increased viral accumulation when inoculated with *Plum pox virus* (PPV)^42^. Therefore, it is not surprising that viral suppressors of PTI have been identified recently, including the PPV coat protein^42^, *Cauliflower mosaic virus* P6^39^ and the movement protein (MP) from *Cucumber mosaic virus*^43^. Although a negative role of the begomovirus NSP in PTI has not been addressed yet, NSP has also been shown to interact with BAK1^12^. Therefore, viral protein-mediated suppression of PTI may impact the positive effect of sequestering the PTI inhibitor NIK1 into an antiviral mechanism during virus infection. These opposing effects of virus infection on PTI may complicate the attempts to prove our model immediately. Recently, we have demonstrated that begomovirus-derived nucleic acids function as viral PAMPs to activate the translational control branch of the NIK1-mediated antiviral signalling^47^. These virus-derived PAMPs may provide the means to eliminate the side effects of viral protein suppressors in studies designed to demonstrate positive and negative interactions between antiviral and antibacterial immunity.

## Methods

### Plant materials and growth conditions

The *Arabidopsis thaliana* Col-0 accession and *nik1-1* (SALK_060808), *nik2-1* (SALK_044363), *nik3-1* (SALK_034037), *fls2* (Salk_141277)*, bak1-4* (Salk_116202) and *sid2-5* (SAIL_112_G09) mutants were obtained from the ABRC and have been described previously^10,38^. The NIK1_C5 and NIK1_C8 complementation transgenic lines, which harbour a 35S:NIK1-GFP construct in the *nik1-1* mutant background, have been described previously^15,18^. Plants were grown in soil (Metro Mix 366) in a growth room at 23 °C, 45% humidity, and 75 µE/m^2^/s^1^ light with a 12-h-light/12-h-dark photoperiod. Four-week-old plants were used for protoplast isolation, pathogen infection, callose deposition, and ROS production assays. Seedlings were germinated on half-strength Murashige and Skoog (MS) plates containing 1% (w/v) sucrose and 0.8% (w/v) agar, grown under the same conditions described above for 10 days, transferred to a six-well tissue culture plate with 2 mL water overnight, and then treated with 100 nM flg22 or H_2_O for the indicated time periods for MAPK and qRT-PCR assays.

### Plasmid constructs for transient expression in protoplasts

The *AtNIK1* open reading frame (ORF) was amplified from *Arabidopsis* Col-0 cDNA using AtNIK1-*Nco*I and AtNIK1-*Stu*I primers (Supplementary Table 1). *Nco*I/*Stu*I-digested *NIK1* ORF was cloned into *Nco*I/*Stu*I previously digested pHBT-FLAG or pHBT-HA vectors. The resulting clones contain *NIK1* fused to HA or to FLAG epitope tags at the C terminus and were confirmed using Sanger sequencing. The point mutations of NIK1km were generated by site-directed mutagenesis with primers as listed in Supplementary Table 1. The generation of T474D and T474A mutants has been previously described^14^.

### Plant transformation

The *bak1-4* and *fls2* lines were transformed with pK7F-NIK1T474D^15^ using the floral dip method. For overexpressing lines, Col-0 were transformed with 2×35S::NIK1-6HA-containing pH7m34GW, generating NIK1-OX8 and NIK1-OX10 lines. Transgene incorporation was monitored with PCR. Transgene expression was confirmed by real-time PCR with NIK1 qPCR primers (Supplementary Table 1). For quantification of gene expression, we used actin as an internal control gene. Independently transformed Col-0 lines expressing the T474D transgene (T474D/Col-0 L9, T474D/Col-0 L10) have been previously described^18^.

### CaLCuV infection and quantification of viral DNA load

*A rabidopsis thaliana* plants at the seven-leaf stage were inoculated with plasmids containing partial tandem repeats of CaLCuV DNA-A^10^ and DNA-B using biolistic delivery. Inoculated plants were transferred to a growth chamber and examined for symptom development (leaf necrosis, chlorosis, leaf epinasty, curly leaves, young leaf death and stunted growth), and infection was confirmed by conventional PCR using CaLCuV DNA-B-specific primers (Supplementary Table 1). In each experiment, 20 plants from each line (Col-0, *nik1-1* and NIK1_C5) were inoculated with 2 µg of tandemly repeated DNA-A plus DNA-B per plant. The course of infection was examined using data from three independent experiments. Viral DNA accumulation was quantified by qPCR using viral DNA-B-specific primers (Supplementary Table 1). Genomic copies of CaLCuV were normalized against an internal control (18S rDNA). For viral DNA quantification, standard curves were prepared using serial dilutions of CaLCuV DNA-B (100 to 10^6^ copies of viral genome per reaction).

### TRV infection and quantification of viral RNA accumulation

Agrobacterium cultures containing TRV-RNA1 (pTRV1) and TRV-RNA2 (pTRV2) T-DNA constructs^48^ were infiltrated onto the lower leaf of four-leaf stage *N. benthamiana* plants using a 1-mL needleless syringe. Infected leaves were confirmed by conventional RT-PCR using TRV-RNA2-specific primers (Supplementary Table 1). TRV was mechanically inoculated to *A. thaliana* Col-0, *nik1-1* and NIK1_C5 lines by rubbing the leaves with sap (K_2_HPO_4_ 0.05 M, pH 7.2, Na_2_SO_3_ 0.01 M) from infected *N. benthamiana* leaves. After 2 weeks of inoculation, viral RNA accumulation was quantified by qRT-PCR using the comparative cycle threshold method, TRV-RNA2-specific primers and actin as an internal control gene.

### Bacterial pathogen infection assay

*Pseudomonas syringae* pv. *tomato* (*Pst*) DC3000 and *P. syringae* pv. *maculicola* (*Psm*) ES4326 strains were cultured overnight at 28 °C in KB medium with 50 µg/ml rifampicin or streptomycin. Bacteria were harvested by centrifugation, washed and adjusted to the desired density with 10 mM MgCl_2_. Leaves of 4-week-old plants were hand infiltrated with the bacterial suspension using a 1-mL needleless syringe and collected to measure bacterial growth. Six leaf discs separated as three repeats were ground in 100 µL H_2_O, and serial dilutions were plated onto TSA medium (1% Bacto tryptone, 1% sucrose, 0.1% glutamic acid, 1.5% agar) with the appropriate antibiotics. Bacterial colony-forming units were counted after incubation at 28 °C for 2 days.

### SA determination

*Arabidopsis* seedlings were germinated and grown for 10 days on half-strength MS plates at 22 °C, 16 h of light. Two hundred and twenty milligrams of fresh seedling were crushed in liquid nitrogen and SA was extracted with a mix of 400 µL of 79% (v/v) isopropanol, 20% (v/v) methanol and 1% (v/v) acetic acid using tungsten beads (30 Hz/s for 2 min). The plant extract was sonicated twice for 10 min at 4 °C. After incubation for 30 min on ice, the extract was purified by centrifugation for 10 min at 13,000 × *g* and 4 °C. The supernatant was filtered (syringe filters, 0.45 mm) and 300 µL of the supernatant were injected into an LC-MS system (ultra-performance liquid chromatography, model 1200 infinity series, coupled to a quadrupole sequential mass spectrometer, model 6430, Agilent). The mobile phase was 2% (v/v) acetonitrile and 98% (v/v) water. The mass of the precursor ion was determined (137/92) and the absolute quantity of free hormone was determined based on calibration curves and standards. Data were analysed using the software Skyline^®^. The ultra-performance liquid chromatography tandem mass spectrometry assays were performed with three biological replicates and the data were analysed with *t* test, *p* < 0.05.

### ROS assay

ROS burst was evaluated with a luminol-based assay. Leaves from 4-week-old *Arabidopsis* plants of each genotype were excised into leaf discs of 0.25 cm^2^ and incubated overnight in 96-well plates with 100 µL of H_2_O to eliminate the wounding effect. H_2_O was replaced with 100 µL reaction solution containing 50 µM luminol and 10 µg/mL horseradish peroxidase (Sigma, USA) with or without 100 nM flg22 supplementation. Measurement was completed with a luminometer (Perkin Elmer, 2030 Multilabel Reader, Victor X3) immediately after adding the solution with 1.5 min interval reading times for a period of 30 min ROS production values from 20 leaf discs per treatment are expressed as the mean relative light units.

### MAPK assays

Ten-day-old seedlings germinated on half-strength MS plates were transferred to 2 mL H_2_O in a 6-well plate to recover overnight and then treated with 100 nM flg22 for 5, 15 or 45 min. Seedlings were ground in IP buffer (20 mM Tris-HCl, pH 7.5, 100 mM NaCl, 1 mM EDTA, 10% glycerol, and 1% Triton X-100), and supernatants were collected after centrifugation. The cleared lysate was incubated with SDS sample buffer and loaded onto 12.5% SDS-PAGE gels. Activated MAPKs were measured by immunoblotting with an α-pErk1/2 antibody (Cell Signaling #9101, USA, 1:2000) and a secondary antibody, goat anti-rabbit IgG-HRP (Santa Cruz, cat # sc-2004, 1:10,000). AtMPK3 and AtMPK6 were directly immunoblotted from total protein extracts with α-AtMPK3 (Sigma-Aldrich, cat # M8318, 1:4000) and α-AtMPK6, (Sigma-Aldrich, cat # 7104, 1:4000).

### RNA isolation and qRT-PCR analysis

For RNA isolation, 10-day-old seedlings grown on half-strength MS plates were transferred to 2 mL H_2_O in a 6-well plate to recover overnight and then treated with 100 nm flg22 for 30 or 90 min. RNA was extracted using TRIzol reagent (Invitrogen, USA) and quantified with a NanoDrop spectrophotometer. Total RNA was treated with RQ1 RNase-free DNase I (Promega, USA) for 30 min at 37 °C and then reverse transcribed with M-MuLV Reverse Transcriptase (NEB, USA). Real-time PCR was performed using iTaq Universal SYBR Green Supermix (Bio-Rad, USA) and a 7900HT Fast Real-Time PCR System (Applied Biosystems, USA). Expression of each gene was normalized to expression of *UBQ10*. The primers used to detect specific transcripts for real-time RT-PCR are listed in Supplementary Table 1.

### Callose deposition assays

The leaves of 6-week-old plants grown in soil were hand inoculated with 0.5 µM flg22 or H_2_O for 24 h. Leaves were then transferred into FAA solution (10% formaldehyde, 5% acetic acid and 50% ethanol) for 12 h and de-stained in 95% ethanol for 6 h. Cleared leaf disks were washed two times with 70% ethanol and then three times with distilled water, followed by incubation in 0.01% aniline blue solution (150 mM KH_2_PO4, pH 9.5) for 15 min. Callose deposits were visualized with a fluorescence microscope. Callose deposits were counted using ImageJ 1.43U software (http://rsb.info.nih.gov/ij/).

### Yeast two-hybrid assay

Different combinations of NIK1K, BAK1K, and FLS2K kinase domains in pGADT7 and pGBKT7 vectors were co-transformed into the yeast AH109 strain as indicated in the figures. Polyethylene glycol/LiAc-mediated yeast transformation was performed according to the Yeastmaker Yeast Transformation System 2 (Clontech) protocol. Protein–protein interactions were tested by growing yeast colonies on synthetic defined medium without histidine, leucine, and tryptophan and supplemented with 1 mM 3-amino-1,2,4-triazole.

### BiFC analysis

For the biochemical complementation assay of YFP, FLS2, BAK1, NIK1 and NIK1-T474D were fused to the N terminus or C terminus of the *YFP* gene. Then, constructs expressing NIK1-cYFP, NIK1-nYFP, BAK1-cYFP, FLS2-nYFP and other indicated fusion proteins were co-agro-infiltrated into tobacco leaves in the presence of the suppressor of silencing HC-Pro in different combinations. YFP fluorescence was observed using confocal microscopy.

### Co-IP assay

*Arabidopsis* protoplasts were transfected with a pair of constructs (empty vector was used as a control) and incubated for 12 h. Samples were collected by centrifugation and lysed with Co-IP buffer (20 mM Tris-HCl, pH 7.5, 100 mM NaCl, 1 mM EDTA, 10% glycerol, 0.5% Triton X-100, and protease inhibitor cocktail) by vortexing. For the Co-IP assay, protein extracts were pre-incubated with protein G agarose beads for 1 h at 4 °C with gentle shaking. Immunoprecipitation was performed with an α-FLAG agarose for 3 h and at 4 °C (α-FLAG M2 Affinity gel, Sigma-Aldrich, Cat # 2220). Beads were collected and washed three times with washing buffer (20 mM Tris-HCl, pH 7.5, 100 mM NaCl, 1 mM EDTA, and 0.1% Triton X-100). Immunoprecipitated and input proteins were analysed by immunoblotting with the antibodies, as indicated in the figures and listed here (α-FLAG M2-peroxidase, Sigma-Aldrich, Cat # A8592, 1:2000; α-HA-peroxidase, Roche, Cat # 12013819001, 1:2000; *α-*GFP, Roche, Cat # 11814460001, 1:1000; goat anti-mouse IgG-HRP, Santa Cruz, Cat # sc-2005, 1:10,000).

### In vitro pull-down and kinase assay

Fusion proteins including GST, GST-NIK1JK (juxtamembrane domain and kinase domain), GST-NIK1JKkm, GST-FLS2JK, GST-BAK1JK, MBP, MBP-FLS2JK and MBP-BAK1JK in vector pGEX4T-1 (Pharmacia) or pMAL-c2 (New England Biolabs) were expressed in the *E. coli* BL21 strain and purified through affinity chromatography with glutathione agarose or amylose resin. For pull-down assay, MBP-FLS2JK and MBP-BAK1JK fusion proteins (tagged with HA) as preys were pre-incubated with 5 μL prewashed glutathione agarose for 0.5 h at 4 °C. The agarose was spin down and the supernatant was collected and incubated with GST or GST-NIK1JK beads for another 1 h. The pull-down proteins were detected with an α-HA antibody by immunoblot. For kinase assay, GST-NIK1JKkm was used as substrate and MBP-FLS2JK or MBP-BAK1JK as the kinase, which were mixed in kinase buffer (20 mM Tris-HCl, pH 7.5, 10 mM MgCl_2_, 5 mM EGTA, 100 mM NaCl, and 1 mM dithiothreitol (DTT)) with 0.1 mM cold ATP and 5 μCi [^32^P]γ-ATP at room temperature for 3 h. The phosphorylation of fusion proteins was analysed by autoradiography after separation with 10% SDS-PAGE.

### Phosphorylation assay

RPL10-GFP was transiently expressed in protoplasts for 16 h, and then the protoplasts were treated with 100 nM flg22 for 3 h. RPL10-GFP was immunoprecipitated with α-GFP antibodies and sepharose-A beads, fractionated by SDS-PAGE and immunoblotted with a α-phosphoserine antibody (α-phosphoserine peroxidase, Sigma-Aldrich, Cat # SAB5200087, 1:5000) and a α-GFP antibody (α-GFP, Roche, Cat # 11814460001, 1:1000; goat anti-mouse IgG-HRP, Santa Cruz, Cat # sc-2005, 1:10,000). Likewise, NIK1-HA was immunoprecipitated from protein extracts prepared from flg22-treated and non-treated NIK1-HA-overexpressing seedlings with α-HA antibodies (α-HA, Thermo Fisher, Cat # 71-5500, 1:50) and sepharose-A beads (Protein A-Sepharose 4B, Thermo Fisher, Cat # 10-1041), fractionated by SDS-PAGE and immunoblotted with a α-phosphoserine antibody (α-phosphoserine peroxidase, Sigma-Aldrich, Cat # SAB5200087, 1:5000); α-phosphotyrosine antibody (α-phosphotyrosine, Thermo Fisher, Cat # 61-5800, 1:2000) and α-phosphothreonine antibody (α-phosphothreonine, Thermo Fisher, Cat # 71-8200, 1:250) and goat anti-rabbit IgG secondary antibody (Thermo Fisher, Cat # 65-6120, 1:10.000).

### RNA-seq method and data analysis

For RNA-seq experiments, we used three biological replicates of a pool of 10-day-old Col-0 and *nik1-1* seedlings and examined differences between Col-0 and *nik1-1* lines using the Deseq2 differential gene expression method^49^. RNA-seq data were obtained using an Illumina Hi-seq 2000. The paired-end 100-bp protocol was used with the following quality filter parameters: 5 bases trimmed at the 3′ and 5′ ends of the reads and a minimum average Phred score of 30. Differentially expressed (DE) genes were stored using SQL tables in the PostgreSQL relational database (http://inctipp.bioagro.ufv.br/arabidopsisnik0/), which listed corresponding log_2_ FC (fold change) and *p* values corrected by false discovery rate (*q* value) for all DE genes. RNA-seq data were then analysed using the eigenvector centrality method^26^ to identify upregulated genes in *nik1-1* plants that represented relevant protein hubs in the plant–pathogen interactome network based on protein–protein and genetic interactions. By considering a fold change >1.5 as the major criterion for eigenvector centrality metrics, *nik1-1* upregulated genes, which were retrieved from the *Arabidopsis* pathogen interactome network database (http://interactome.dfci.harvard.edu/A_thaliana), were classified by gene ontology categories.

### Rearrangement of the LRR-RLK interaction network

The RLK extracellular interactome network (CSI^LRR^) was obtained from Smakowska-Luzan et al. ^28^. The network was created by Cytoscape 3.6.1^50^. The approximation of maximum cut algorithm was adapted from Goemans–Williamson Algorithm^51^.

### Reporting summary

Further information on research design is available in the Nature Research Reporting Summary linked to this article.

## Supplementary information

Supplementary Information

Reporting Summary

Source Data

## Data Availability

The authors declare that the data supporting the findings of this study are available within the paper and in Supplementary Information files. The source data underlying Figs. 1a, b, 1d, 1f, 1h, i, 2a, e, 2g, 3c, j, 4a, i, 5a, g, 5i, l, 6a–i and Supplementary Figs. 1d–g, 2b, c, 3b–h, 4a–i, 5a–f, 8a–f, 9b–f are provided as Source Data files. The pipeline of RNA-seq analysis and data can be found at http://inctipp.bioagro.ufv.br/arabidopsisnik0 and SRA accession number PRJNA573716.
