# Supplementary material for: The receptor-like kinase NIK1 targets FLS2/BAK1 immune complex and inversely modulates antiviral and antibacterial immunity
Source: Nat Commun. 2019 Nov 1;10:4996. doi: 10.1038/s41467-019-12847-6 (PMC6825196; doi:10.1038/s41467-019-12847-6)
Supplement: Supplementary file 1 — Supplementary Information [file 41467_2019_12847_MOESM1_ESM.pdf]

Supplementary Information (Supplementary Figures / Table)

The receptor-like kinase NIK1 targets FLS2/BAK1 immune complex and inversely modulates antiviral and antibacterial immunity

Li et al, 2019

Supplementary Figure 1

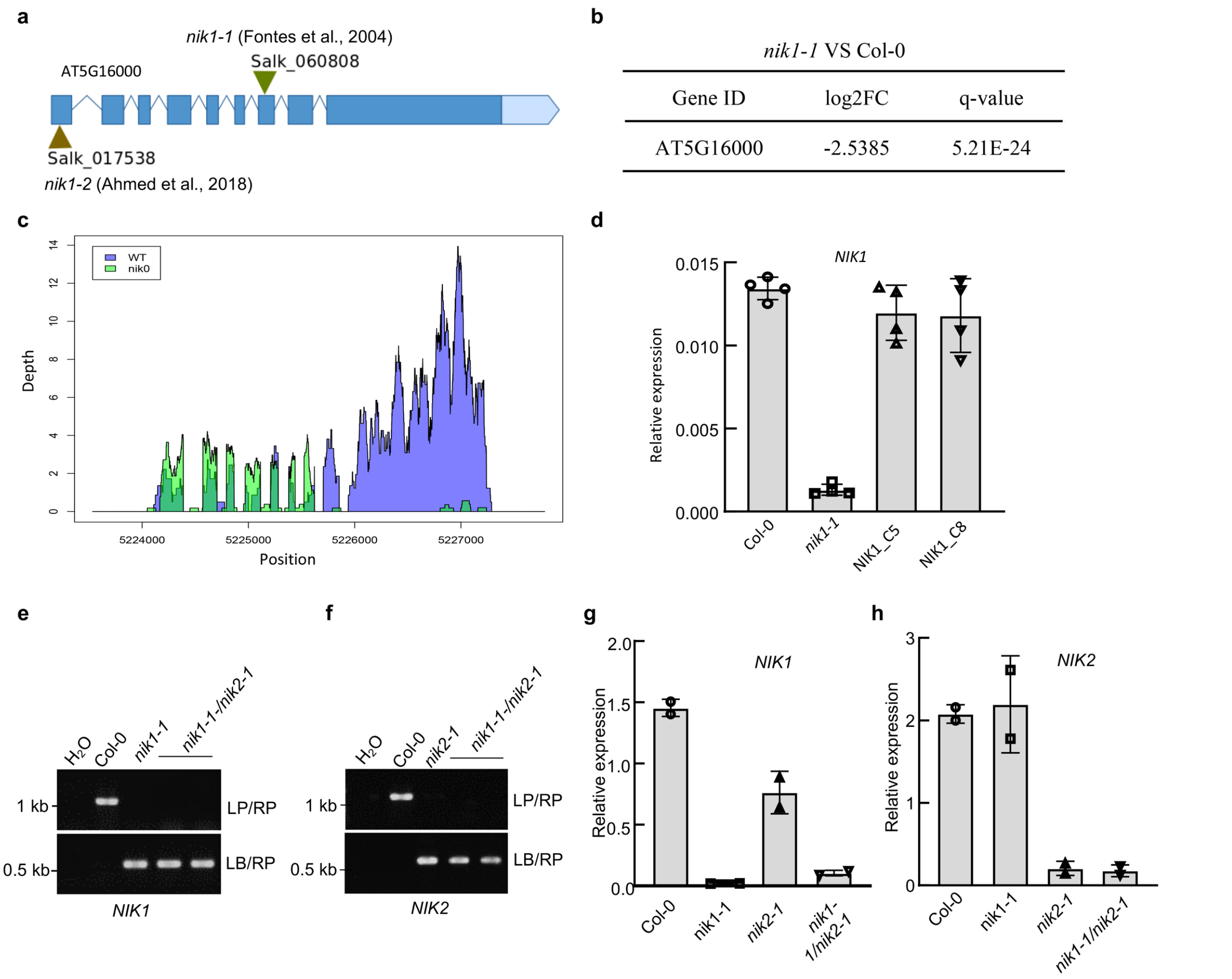

Supplementary Fig. 1 Characterization of *nik1-1* and *nik2-1* T-DNA insertional mutants. a Schematic structure of the annotated AT5G1600 (*NIK1)* locus and T-DNA insertional mutants. Blue boxes indicate exons and curve lines represent introns. Triangles indicate the positions of the T-DNA insertions in the *nik1-1* and *nik1-2* mutants. **b** *NIK1* relative expression level in *nik1-1* mutant compared to Col-0 as determined by RNA-seq. **c** High throughput sequencing reads mapped to *NIK1* in *nik1-1* mutant. **d** NIK1 transcript accumulation in *nik1-1* and in complemented lines (R4 generation). Total RNA was isolated from Col-0, *nik1-1*, *35S:NIK1-GFP/nik1-1* lines NIK1_C5 and NIK1_C8 leaves and the expression of *NIK1* was monitored by quantitative RT-PCR. Mean ± 95% confidence intervals based on bootstrap resampling replicates of three independent experiments. **e** Genotyping of *nik1-1/nik2-1* double mutant for a T-DNA insertion in *NIK1*. PCR was carried out with genomic DNA using either the LB/*NIK1*_RP primers for T-DNA or *NIK1*_LP/ NIK1_RP primers for gDNA. **f** *G*enotyping of *nik1-1/nik2-1* double mutant for a T-DNA insertion in *NIK2*. PCR was carried out with genomic DNA using either the LB/*NIK2*_RP primers for T-DNA or *NIK2*_LP/ *NIK2*_RP primers for gDNA. **g** and **h** Relative *NIK1* or *NIK2* expression levels in double mutants. Total RNA was isolated from the indicated lines, and the relative gene expression was quantified by qRT-PCR and *UBQ10* as an internal control. The values are mean ± SE (n=3).

Supplementary Figure 2

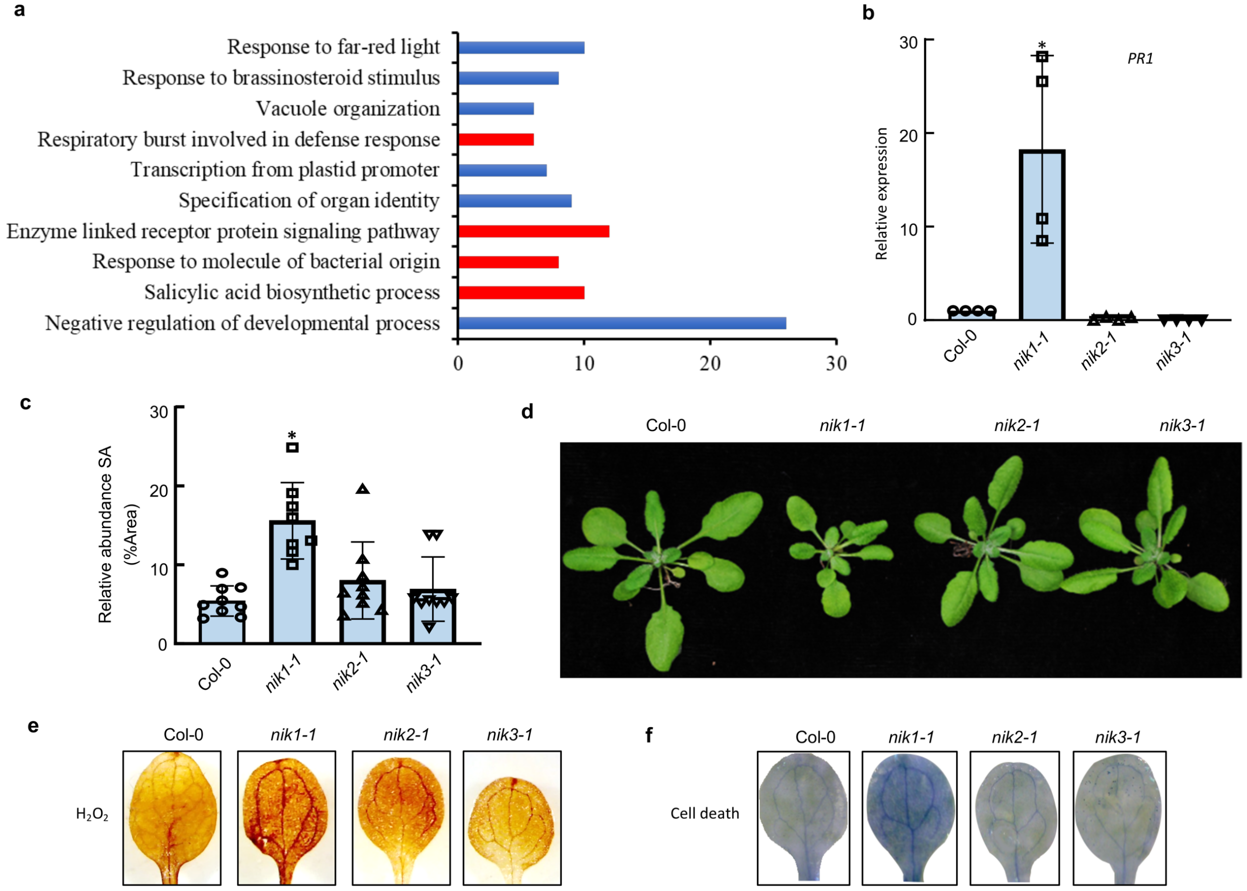

Supplementary Fig. 2 Constitutive activation of SA-related defence responses in the *nik1-1* knockout line. a Functional categorization of up-regulated genes in *nik1-1* mutant by RNA-seq. The bar graphs illustrate the distribution of up-regulated genes that represent relevant hubs across functional categories as defined by the Gene Ontology (GO) biological process. The numbers represent the percentage of genes up-regulated in *nik1-1* relative to the WT control that represent relevant hubs in each category. **b** PR1 is constitutively up-regulated in the *nik1-1* mutant. Total RNA was isolated from Col-0, *nik1-1*, *nik2-1* and n*ik3-1* lines, and the relative PR1 expression was quantified by qRT-PCR and UBQ10 as an internal control. The mean values are relative to the wild-type (n=3). Asterisks indicate significant differences ($p < 0.05$). **c** Enhanced accumulation of SA in *nik1-1* plants. The relative SA content was determined in leaf extracts from the Col-0 and NIKs mutants. Values are the mean ± SD of three replicates. Statistical significance is indicated by asterisks (t-test, $p < 0.05$). (**d**) Plant growth phenotype of different *NIKs* mutants. The Col-0, *nik1-1*, *nik2-1* and *nik3-1* plants grown under normal conditions. Images were taken four weeks post gemination. (**e**) H_2O_2 production in Col-0 and *niks* mutants. DAB staining for H_2O_2 production is shown in the leaves of knockout lines and Col-0. (**f**) Cell death in Col-0 and *niks* mutants. Trypan blue staining for cell death is shown in leaves of knockout lines and Col-0.

Supplementary Figure 3

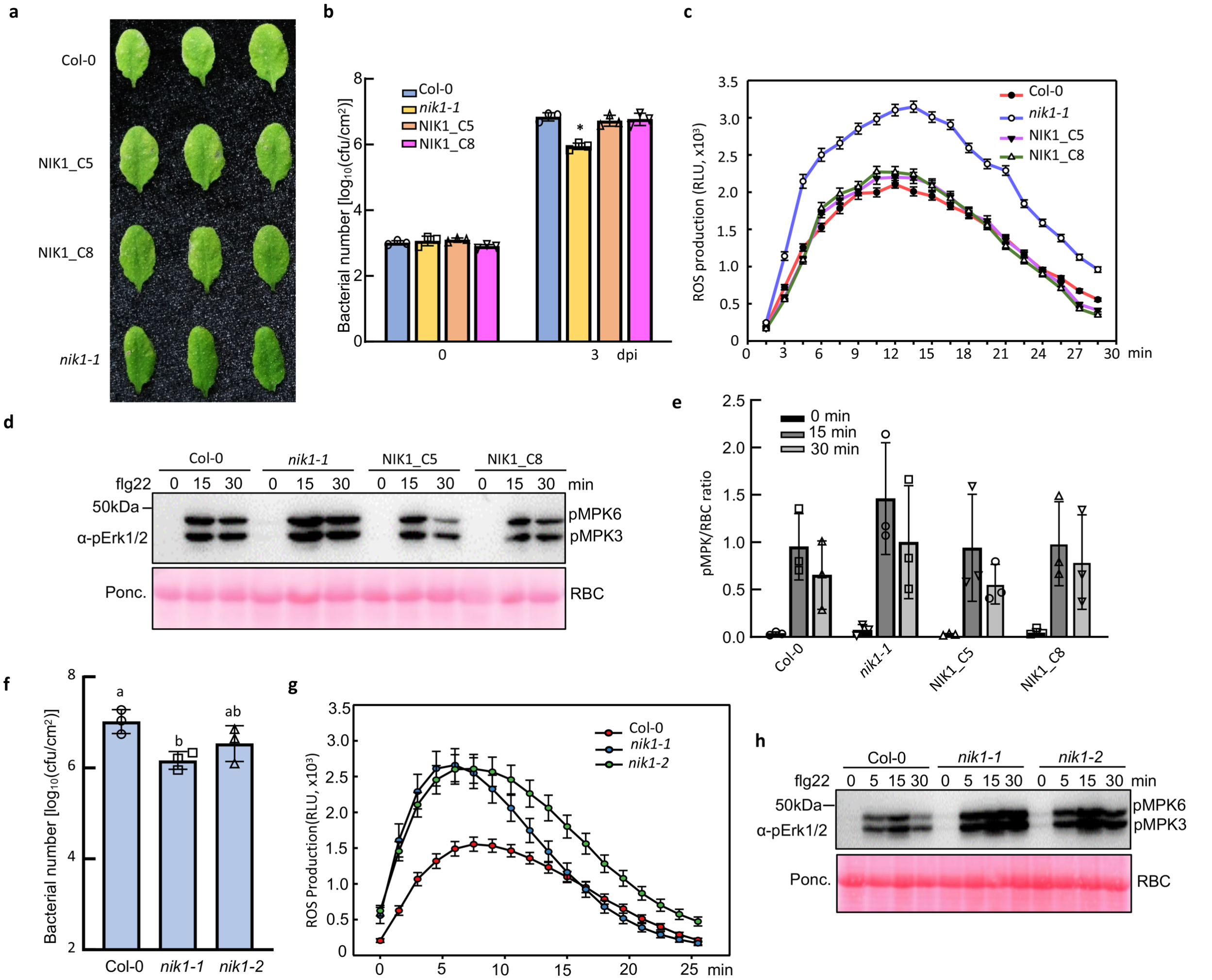

Supplementary Fig. 3 NIK1 reintroduction restores the *nik1-1* mutant disease phenotype. a Disease symptom caused by *Pst* DC3000 infection. Images were taken at 4 dpi. **b** Bacterial growth of *Pst* DC3000 4 days post inoculation. Four-week-old plants Col-0, *nik1-1* and *nik1-1*-complementing lines were hand-inoculated with bacterial suspensions of *Pst* DC3000 at a density of $5x10^5$ cfu/mL, and bacterial populations were quantified at 0 and 4 dpi. **c** Flg22-induced ROS burst in *nik1-1*-complementing lines. Leaf discs from 5-week-old plants were treated with water or 100 nM flg22 and the relative light units (RLU) were detected. Values are the mean ± SE (n>15). **d** Flg22-induced MAPK activation in *nik1-1*-complementing lines. Ten-day-old seedlings of the indicated lines were treated with 100 nM flg22. MAPK activation was detected with an α-pERK antibody (top panel). Total protein input was evaluated by Ponceau S staining for Rubisco (RBC) (bottom panel). **e** Quantitative data for MAPK activation in (**d**). IB images were analysed by ImageJ and the values are the mean ± SD of three replicates. **f** Growth of *Pst* DC3000 four days post-infection. Four-week-old plants of Col-0, *nik1-1*, *and nik1-2* mutants were hand-inoculated with *Pst* DC3000. **g** Flg22-induced ROS burst in WT, *nik1-1* and *nik1-2* plants. Leaf discs from 5-week-old plants were treated with water or 500 nM flg22 for 30 min, and the relative light units (RLU) were detected. Values represent the mean ± SE (n>10). **h** Flg22-induced MAPK activation in WT, *nik1-1* and *nik1-2* plants. MAPK activity was assayed as described in Fig. 2b.

Supplementary Figure 4

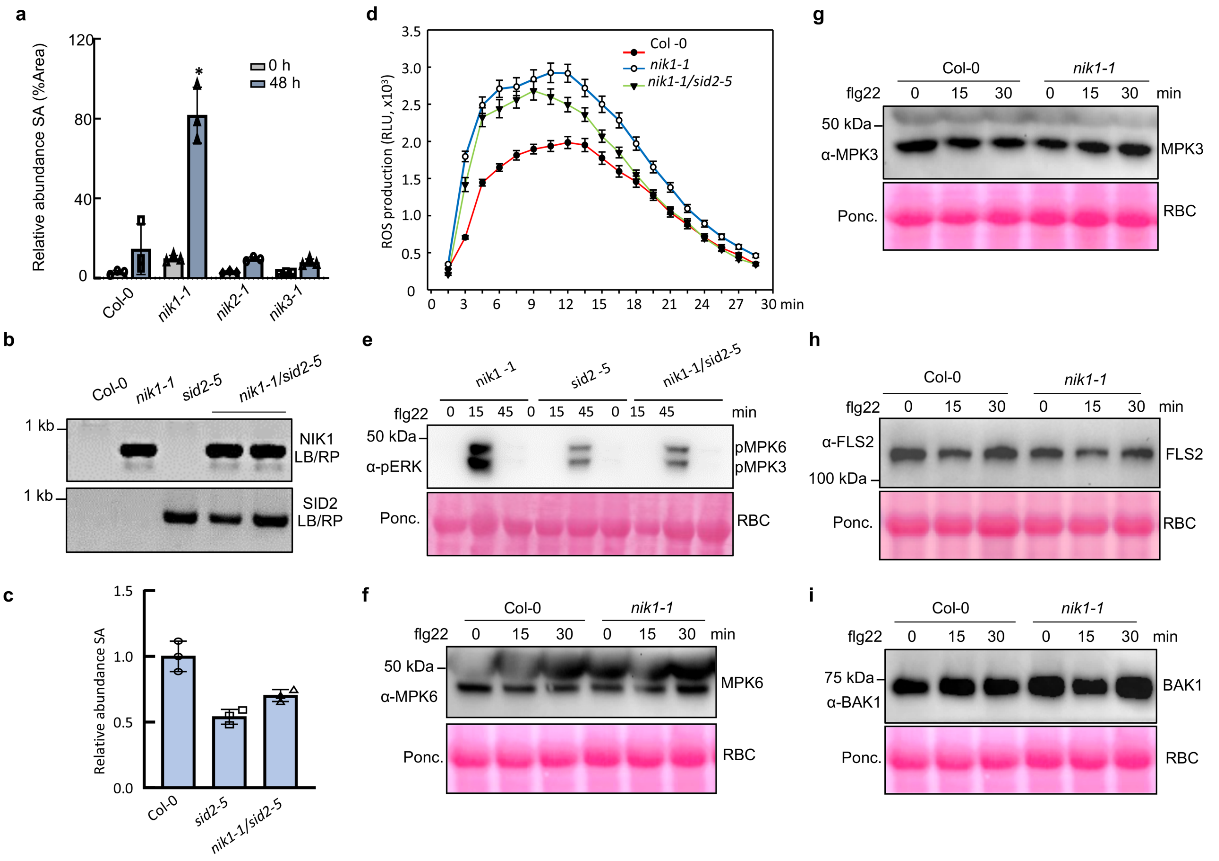

Supplementary Fig. 4 The resistant phenotype of *nik1-1* mutants is independent of high SA accumulation. a Relative SA accumulation level in Col-0 and *nik* mutants post bacterial infection. The bars indicate 95% confidence intervals based on the t test ($p < 0.05$, n = 3). Asterisks indicate significant differences relative to the same treatment in Col-0. **b** Genotyping *nik1-1/sid2-5* double mutants. PCR was carried out with genomic DNA using either the LB/*NIK1*_RP primers for *NIK1* T-DNA or LB/*SID2*_RP primers for *SID2* T-DNA. **c** Relative SA accumulation level in *nik1-1*, *sid2-5* and *nik1-1/sid2-5*. The relative content of salicylic acid (SA) was determined in leaf extracts from four-week-old plants. The error bars indicate 95 % confidence intervals based on t-tests ($p < 0.05$, n = 3). **d** Total ROS production in response to flg22 treatment. Leaf discs from 5-week-old plants were treated with water or 100 nM flg22 and the relative light units (RLU) were detected. Values are the mean ± SE (n>15). **e** Flg22-induced MAPK activity in *nik1-1*, *sid2-5* and *nik1-1/sid2-5*. **f** and **g** Endogenous MPK6 and MPK3 protein levels in *nik1-1* mutant. Ten-day-old seedlings of indicated lines were treated with 100 nM flg22 for 15 and 30 min. MPK protein levels were detected by α-MPK6 and α-MPK3 antibodies. **h** Endogenous FLS2 protein levels in *nik1-1* mutants. FLS2 protein levels were detected with an α-FLS2 antibody (top panel). Total protein input was showed by Ponceau S staining for RBC (bottom panel). **i** Endogenous BAK1 protein levels in *nik1-1* mutants. BAK1 protein levels were detected using an α-BAK1 antibody.

Supplementary Figure 5

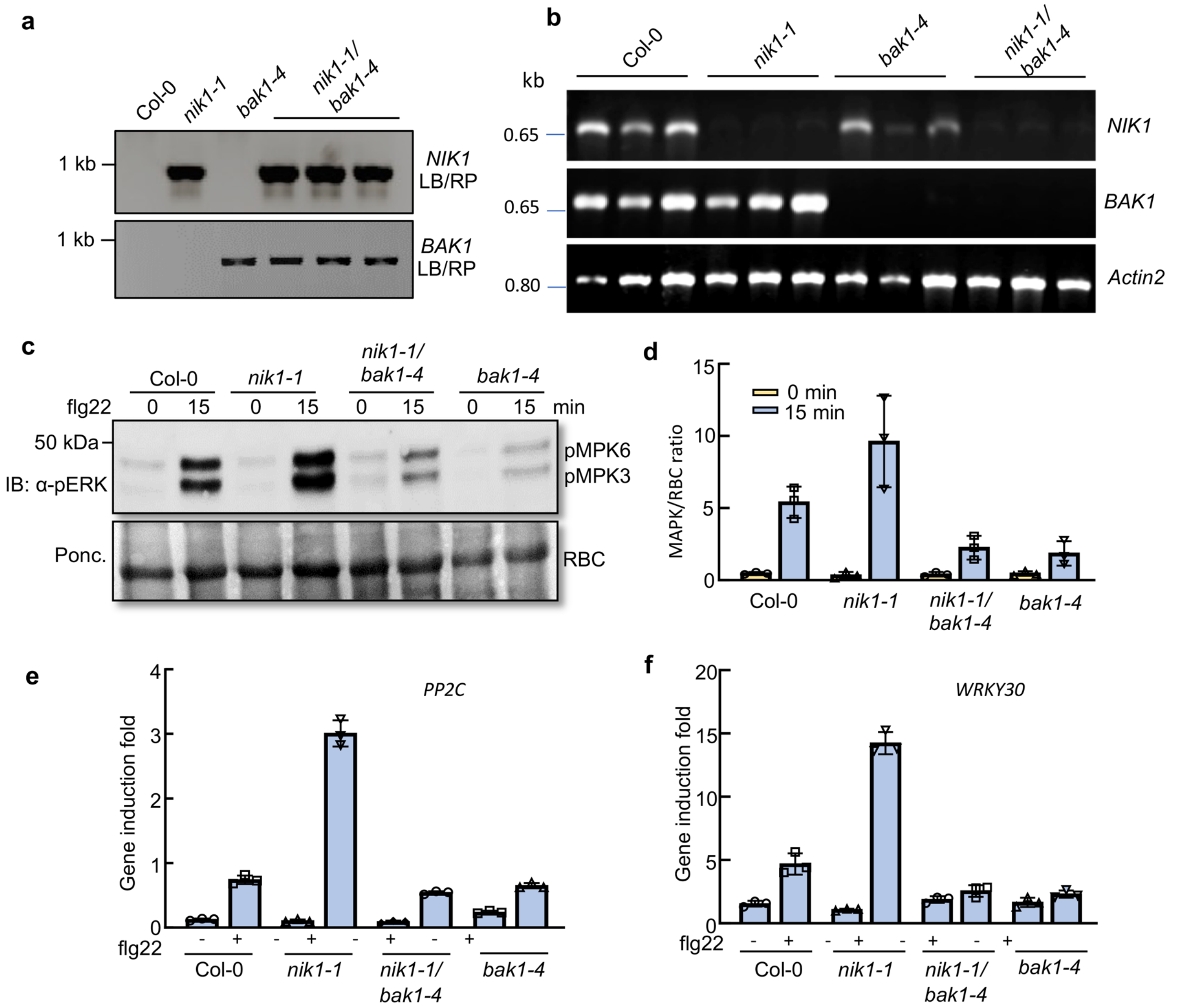

Supplementary Fig. 5 BAK1 is required by NIK1 to suppress flg22-mediated immunity. a Genotyping analysis of *nik1-1/bak1-4* double mutants. PCR was performed with genomic DNA using either the LB/*NIK1*_RP primers for *NIK1* T-DNA or LB/ *BAK1*_RP primers for *BAK1* T-DNA. **b** Gene expression levels in *nik1-1/bak1-4* double mutant. RT-PCR analysis of *NIK1* and *BAK1* gene expression with cDNA from the indicated plants. *ACTIN2* served as an internal control. **c** Flg22-induced MAPK activation in Col-0, *nik1-1*, *bak1-4* and *nik1-1/bak1-4*. Ten-day-old seedlings from half MS medium were treated with 100 nM flg22 for 15 min and MAPK activity was detected by immunoblotting (IB) with an α-pERK antibody (top panel). Total input proteins were showed by Ponceau S staining for RBC (bottom panel). **d** Quantitative data for MAPK activation in (**c**). IB signals were analysed using ImageJ, and the values represent the mean ± ED of three repeats. The error bars indicate 95 % confidence intervals based on t-tests ($p < 0.05$). **e** and **f** *PP2C* and *WRKY30* induction in response to flg22 treatment. Total RNA was extracted from 10-day old seedlings with or without flg22 treatment for 30 min. qPCR was performed with cDNA from the indicated lines. Values are the mean ± SD of three repeats.

Supplementary Figure 6

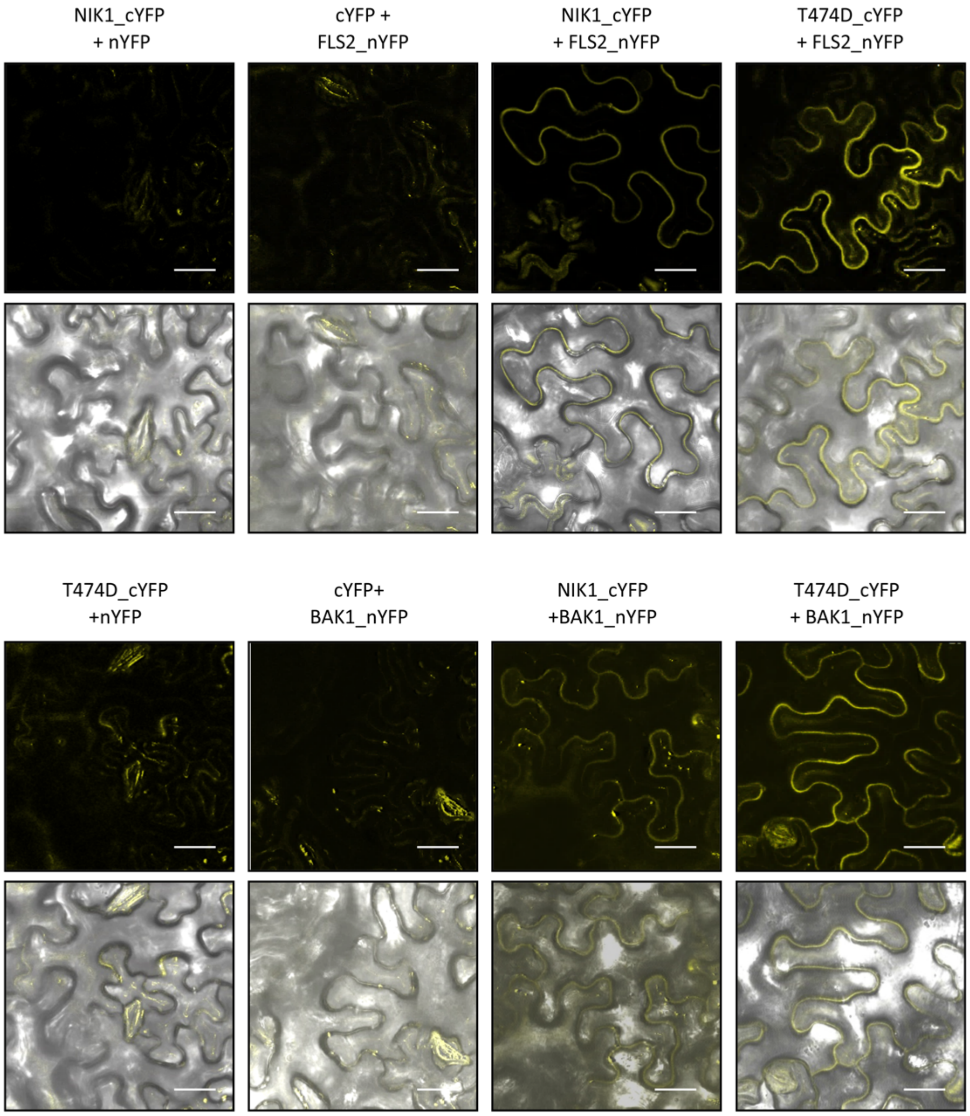

Supplementary Fig. 6 In vivo interactions between NIK1-T474D and FLS2 or BAK1 by BiFC assays. Different combinations of nYFP, NIK1-cYFP, T474D-cYFP, FLS2-nYFP or BAK1-nYFP were co-expressed in *N. bethamiana* leaf through agrobacterial-infiltration in the presence of HC-Pro suppressor. Fluorescence (YFP) signal and bright field images were taken of epidermal cells of tobacco with a Confocal microscopy. Scale bars = 20 μm.

Supplementary Figure 7

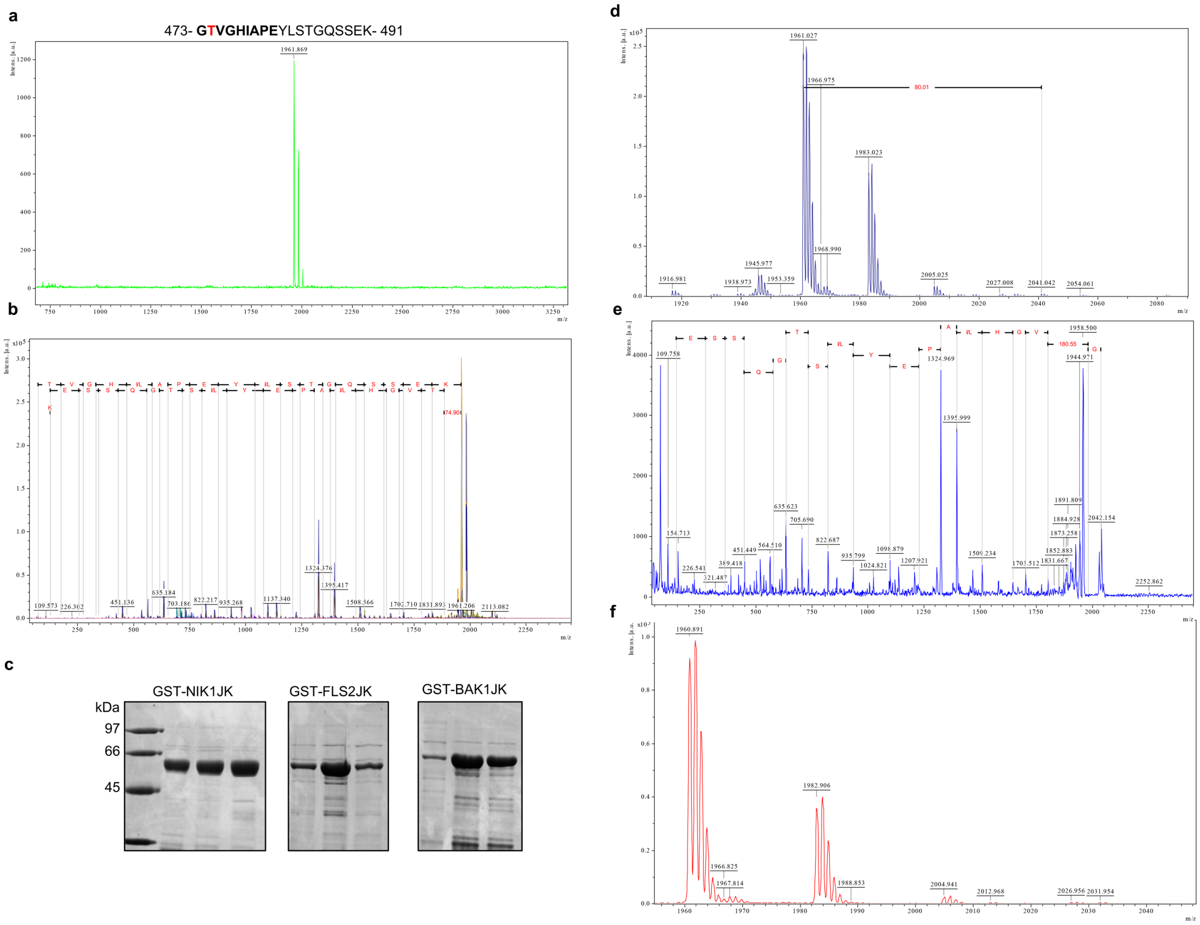

Supplementary Fig. 7 The Thr474 residue is one important site of NIK1 phosphorylation activity. a and **b** Mass spectrum analysis confirms the *in vitro* synthesized Thr474-containing NIK1 peptide sequence from the NIK1 activation loop. **c** *E. coli*-produced GST fusion proteins of NIK1JK (juxtamembrane domain and kinase domain), BAK1JK and FLS2JK were separated by SDS-PAGE and Coomassie blue-stained. **d** and **e** NIK1 autophosphorylation on Thr474 residue. MS spectrum of the *in vitro* synthesized peptide used as substrate in an *in vitro* kinase assay with GST-NIK1JK. The MS/MS spectrum of the phosphorylated peptide is shown in (**e**). **f** FLS2 does not phosphorylate NIK1 Thr474 in an *in vitro* kinase assay. The substrate peptide molecular weight monitored by MS spectrum from reaction with FLS2JK.

Supplementary Figure 8

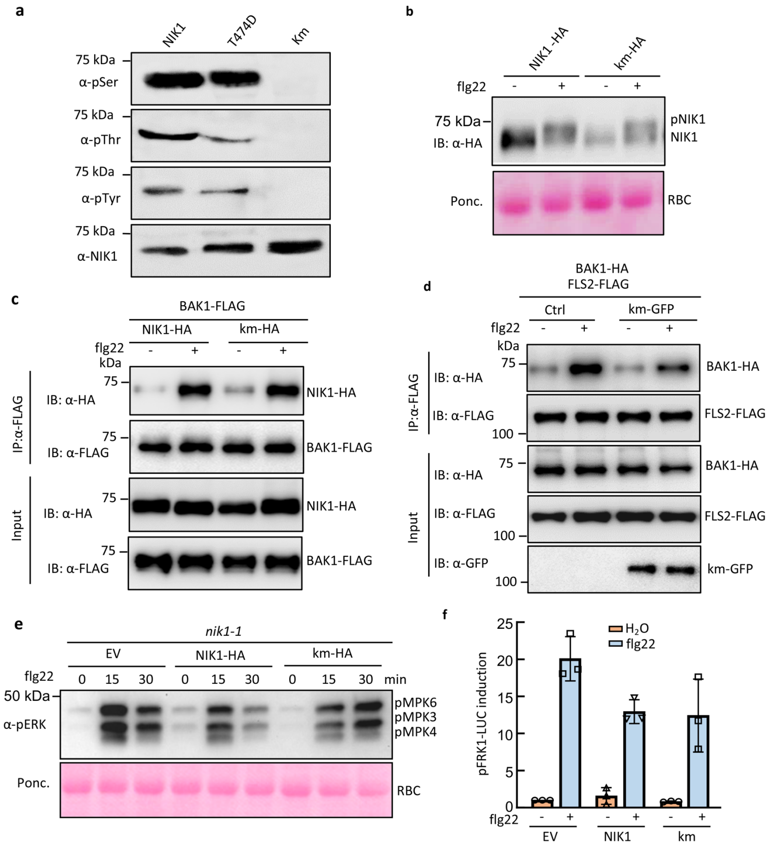

Supplementary Fig. 8 NIK1 kinase activity is dispensable for the NIK1 negative regulatory role in the FLS2 signalling pathway. a Kinase activity of different NIK1 variants. NIK1 phosphorylation levels were detected by immunoblotting with α-phosphoserine (α-pSer), α-phosphotheronine (α-pThr) antibodies or an α-phosphotyrosine (α-pTyr) antibody. NIK1 proteins were detected using an α-NIK1 antibody as loading controls. **b** Flg22-induced mobility shift of NIK1 and NIK1 kinase mutant (km). Protoplasts were transfected with NIK1-HA or NIK1km-HA. Flg22 treatment was performed for 15 min before samples collection. Total proteins were showed by Ponceau S staining for RBC (bottom panel). **c** Flg22-induced association of BAK1 with NIK1 and NIK1km. BAK1-FLAG was co-expressed with NIK1-HA or NIK1km-HA in *Arabidopsis* protoplasts treated with 100 nM flg22. Co-IP assays were performed with α-FLAG agarose beads and with α-HA or α-FLAG for IB (top two panels). The input control is shown on the bottom two panels. **d** Flg22-induced BAK1/FLS2 complex formation was inhibited by NIK1km. BAK1-HA and FLS2-FLAG were co-expressed with NIK1km-GFP in *Arabidopsis* protoplasts treated with 100 nM flg22 for 10 min. Co-IP was performed as in (**c**). **e** Flg22-induced MAPK activation in *nik1-1* protoplasts expressing empty vector (EV), NIK1-HA or NIK1km-HA. Protoplasts were treated with 100 nM flg22 for 15 and 30 min, and MAPK activity was detected by IB with an α-pERK antibody (top panel). Total input proteins were showed by Ponceau S staining for RBC (bottom panel). **f** NIK1 and NIK1km suppress pFRK1::LUC induction. The pFRK1::LUC was co-transfected with vector control or NIK1 variants in protoplasts. UBQ10-GUS was included as a control and the luciferase activity was normalized with GUS activity. The data are shown as the mean±SD from three independent biological replicates.

Supplementary Figure 9

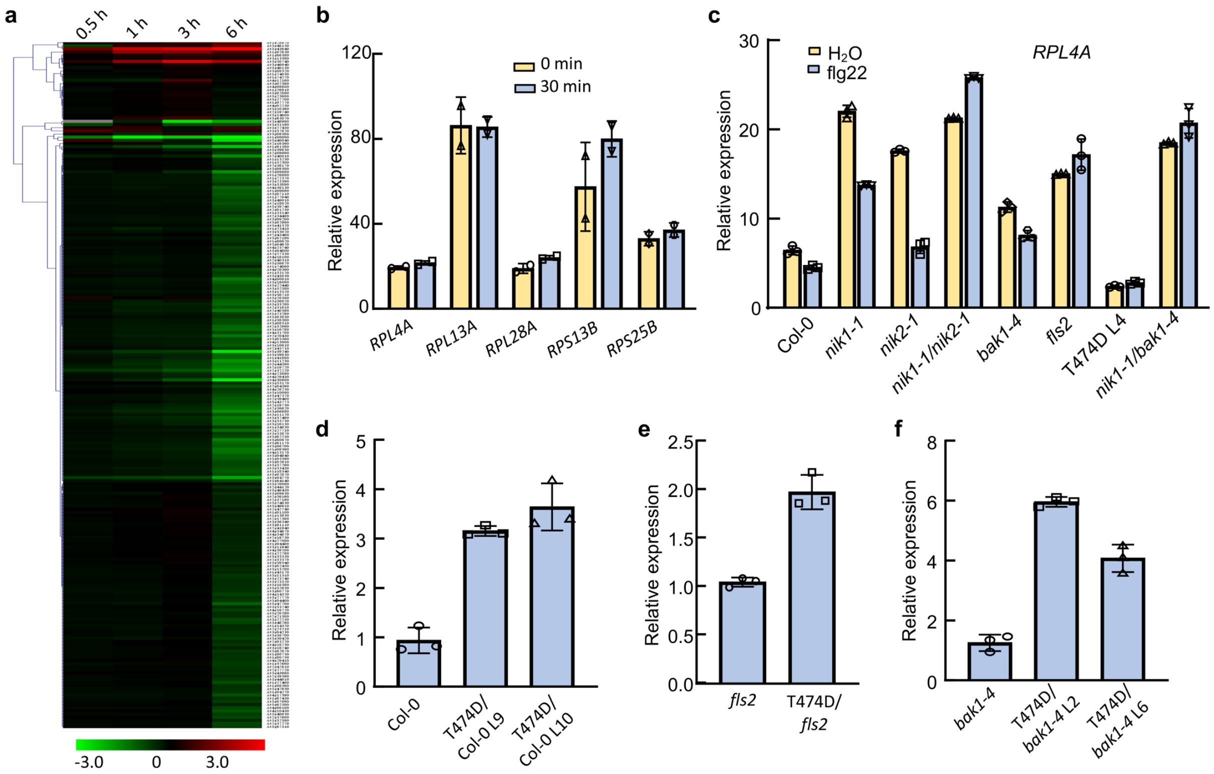

Supplementary Fig. 9 Flg22-induced suppression of ribosomal gene expression in a NIK1 and FLS2/BAK1 dependent manner. a Expression profiles of the *Arabidopsis* ribosomal protein gene family in response to flg22 treatment. Heatmap was generated based on the fold change after $\log_2$ normalization with Mev software. **b** Transcript accumulation of selected RB genes 30 min after flg22 treatment. Gene expression of the indicated RB genes was determined by quantitative RT-PCR and *ACTIN2* as an internal control. **c** Flg22-induced suppression of *RPL4A* expression in different mutants. qRT-PCR analysis of *RPL4A* gene expression was performed with cDNA from indicated plants treated with or without flg22 treatment. *ACTIN2* served as an internal control. **d**, **e** and **f** NIK1 expression levels in Col-0, *fls2*, *bak1-4* and T474D-overexpressing lines as determined by qRT-PCR. The respective 95% confidence interval limits were estimated based on bootstrap resampling replicates of three independent (n=3) experiments and three technical repeats.

Supplementary Table 1

**Supplementary Table 1- Primers used in methods**		
**Primers for qRT-PCR**		
	Fwd Primer	Rvs Primer
NIK1 qPCR	AGGCACGGTGGGTCACATT	TCCCGAAGCCAAAAACATCT
NIK2 qPCR	CCAATGGCAGTGTCGCTTCT	TGCTCCTAATGCTATTCGCTTTC
FRK1 qPCR	GCCAACGGAGACATTAGAG	CCATAACGACCTGACTCAT
WRKY30 qPCR	AGCCAAATTTCCAAGAGGAT	GCAGCTTGAGAGCAAGAATG
PP2C qPCR	CGTGTTGGGGATTGATTCG	AGAGCTCGGGCGGTTATG
PR1 qPCR	GTTAGCGAGAAGGCTAACTAC	CATCCGAGTCTCACTGACTTTC
NHL10	TTCCTGTCCGTAACCCAAAC	CCCTCGTAGTAGGCATGAGC
PHI1	TTGGTTTAGACGGGATGGTG	ACTCCAGTACAAGCCGATCC
UBQ10 qPCR	AGATCCAGGACAAGGAAGGTATTC	CGCAGGACCAAGTGAAGAGTAG
ACTIN2 qPCR	ATGTCGTGAGCCATCCTGTC	ACACCGGATTCGTGCGGCATAGAAG A
At2G19730 RPL28A	AAGCACTCTGGTCTTGCAAACA	GTGGTGGCGAGCACAACAG
At2G21580 RPS25B	CCGATCGTTACTCCGTCGAA	AGATTTGGCCGGCTTTGAT
At3G07110 RPL13A	GGCTGATCCAGAGCTGAGTGAAA	GTGGTGACGAGCATCAACCA
At3G09630 RPL4A	GTCACTGTTAGCTGAAGCACAGAG A	CCTCCTTGGTAACGGTTTTCC
At4G00100 RPS13B	TCAGGCTCATCTTGGTTGAGA	CAGACGGGAGGGAGCTTCTT
qRT-TRV2	TGCAGTGGCGGTGTTACAA	GTCGAGCCAGTGTTCGCCTT
**Primers for qPCR**		
CaLCuV DNA-B qPCR	GGGCCTGGGCCTGTTAGT	ACGGAAGATGGGAGAGGAAGA
18S rDNA qPCR	TAATTTGCGCG CCTGCTGCC	TGTGCTGGCGACGCATCATT
**Primers for RT-PCR**		
NIK1 RT	AAAAAGCAGGCTTCACAATGGGA GCTGCAAGAGGG	AGA AAG CTG GGT CTC ATC TAG GACCAG AGA GCT C
NIK2 RT	CCAATGGCAGTGTCGCTTCT	TGCTCCTAATGCTATTCGCTTTC
BAK1 RT	AAAAAGCAGGCTTCACAATGGACC CAGAAGTTCA	AGAAAGCTGGGTCTTCATTAAAGCA TTCTTACAAC
ACTIN2 RT	GGCTGGATTTGCAGGAGATG	ACCATCACCAGAATCCAGCAC
**Primers for point mutations**		
NIK1Km	GTGGTTGCAGTGGAAAGGCTTAAA GAT	ATCTTTAAGCCTTTCCACTGCAACC AC
**Primers for conventional PCR**		
CaLCuV DNA-B	GGCGTGGGGTATCTTACTC	GACAT AGCATCGGACATCC
**Primers for cloning**		
AtNIK1- NcoI	CCATGCCATGGAGAGTACTATTGTT ATGATG	
AtNIK1- StuI	GAAGGCCTTCTAGGACCAGAGAGC TCCATTG	

**Primers for genotyping**		
LB b1.3	ATTTTGCCGATTTCGGAAC	
NIK1 LP	GATACACAAGCCCTCTTGCAG	
NIK1 RP	TGTTGTGTATCATCAGGAGGC	
NIK2 LP	CCAAAGAAGAAAACCAAAGCC	
NIK2 RP	AGAGAAGCTCCAAGCCAAAAC	
BAK1 LP	CATGACATCATCATCATTCGC	
BAK1 RP	ATTTTGCAGTTTTGCCAACAC	
SID2 LP	AAGACACAATCCGATTTGCTG	
SID2 RP	TCTGATGGATCTCCAATCGTC	
