# Supplementary material for: The receptor-like kinase NIK1 targets FLS2/BAK1 immune complex and inversely modulates antiviral and antibacterial immunity
Source: Nat Commun. 2019 Nov 1;10:4996. doi: 10.1038/s41467-019-12847-6 (PMC6825196; doi:10.1038/s41467-019-12847-6)
Supplement: Supplementary file 2 — Reporting Summary [file 41467_2019_12847_MOESM2_ESM.pdf]

nature research

Corresponding author(s): Elizabeth P. B. Fontes
DBPR NCOMMS.17.03524B

Last updated by author(s): Sep 24, 2019

Reporting Summary

Nature Research wishes to improve the reproducibility of the work that we publish. This form provides structure for consistency and transparency in reporting. For further information on Nature Research policies, see Authors & Referees and the Editorial Policy Checklist.

Statistics

For all statistical analyses, confirm that the following items are present in the figure legend, table legend, main text, or Methods section.

n/a	Confirmed	
☐	☒	The exact sample size (n) for each experimental group/condition, given as a discrete number and unit of measurement
☐	☒	A statement on whether measurements were taken from distinct samples or whether the same sample was measured repeatedly
☐	☒	The statistical test(s) used AND whether they are one- or two-sided<br>*Only common tests should be described solely by name; describe more complex techniques in the Methods section.*
☒	☐	A description of all covariates tested
☐	☒	A description of any assumptions or corrections, such as tests of normality and adjustment for multiple comparisons
☐	☒	A full description of the statistical parameters including central tendency (e.g. means) or other basic estimates (e.g. regression coefficient) AND variation (e.g. standard deviation) or associated estimates of uncertainty (e.g. confidence intervals)
☐	☒	For null hypothesis testing, the test statistic (e.g. F, t, r) with confidence intervals, effect sizes, degrees of freedom and P value noted<br>*Give P values as exact values whenever suitable.*
☒	☐	For Bayesian analysis, information on the choice of priors and Markov chain Monte Carlo settings
☒	☐	For hierarchical and complex designs, identification of the appropriate level for tests and full reporting of outcomes
☒	☐	Estimates of effect sizes (e.g. Cohen's d, Pearson's r), indicating how they were calculated

Our web collection on statistics for biologists contains articles on many of the points above.

Software and code

Policy information about availability of computer code

Data collection	No software used.
Data analysis	For the RNA-Seq, we used the public available Deseq2 differential gene expression (DGE) method (Love, M. I., Huber, W. & Anders, S. Moderated estimation of fold change and dispersion for RNA-seq data with DESeq2. Genome Biol 15, 550, doi:10.1186/s13059-014-0550-8, 2014).RNA-seq data were then analysed using the eigenvector centrality method (Ahmed H, Howton TC, Sun YL, Weinberger N, Belkhadir Y, Mukhtar MS. Network biology discovers pathogen contact points in host protein-protein interactomes. Nat Commun 9, 2018) to identify upregulated genes in nik1-1 plants that represented relevant protein hubs in the plant-pathogen interactome network based on protein-protein and genetic interactions. The interactome was generated using Cytoscape software.

For manuscripts utilizing custom algorithms or software that are central to the research but not yet described in published literature, software must be made available to editors/reviewers. We strongly encourage code deposition in a community repository (e.g. GitHub). See the Nature Research guidelines for submitting code & software for further information.

Data

Policy information about availability of data

All manuscripts must include a data availability statement. This statement should provide the following information, where applicable:

- Accession codes, unique identifiers, or web links for publicly available datasets
- A list of figures that have associated raw data
- A description of any restrictions on data availability

The pipeline of RNA-seq analysis and data can be found at http://inctipp.bioagro.ufv.br/arabidopsisnik0 and SRA accession PRJNA573716 [https://www.ncbi.nlm.nih.gov/sra/PRJNA573716]. List of figures that have associated raw data: Figs. 1a-b, 1d, 1f, 1h-i, 2a, 2c-e, 2g, 3f, 3h, 3j, 4b, 4d-f, 4h-i, 5j, 5l, 6c-i and Supplementary Figs. 1d, 1g, 2b-c, 3b-c, 3e-g, 4a, 4c-d, 5d-f, 8f, 9b-f. The source data underlying those figures are provided as Source Data files.

Field-specific reporting

Please select the one below that is the best fit for your research. If you are not sure, read the appropriate sections before making your selection.

☒ Life sciences ☐ Behavioural & social sciences ☐ Ecological, evolutionary & environmental sciences

For a reference copy of the document with all sections, see nature.com/documents/nr-reporting-summary-flat.pdf

Life sciences study design

All studies must disclose on these points even when the disclosure is negative.

Sample size	The size of the RNA-Seq experiment was determined based on previous experiments. We used three biological replicates of a pool of 10-day-old Col-0 and nik1-1. Differential gene expression methods were optimized to small number of replicates, then we used three biological replicates per treatment. Also the number of three biological replicates of each gene in qRT-PCR has been reported to be sufficient in the analysis of the confidence intervals based on bootstrap. For in vivo experiments (protein-protein interactions, phosphorylation assays, MAPK assay, ROS assay, bacterial growth, etc), we performed at least three biological repeats. Transgene insertion and expression were confirmed by PCR on leaf DNA and qRT-PCR on RNA leaves of transgenic lines. Each biological replicate was constituted by the pooled fraction of RNA from three plants. In addition, we took extra measures to ensure the reproducibility of our results by using two or more independent approaches to address the same question. For example, protein-protein interactions, protein phosphorylation, activation of PTI, activation of NIK1-mediated antiviral signaling.
Data exclusions	No data were excluded unless the positive control failed. For example, for quantification of viral RNA and DNA we used only inoculated plants that had been confirmed to be infected. The viral infection in CaLCuV-inoculated plants was confirmed by PCR using leaf DNA as template and viral DNA genomic components (DNA-A and DNA-B)-specific primers. For TRV, we used RT-PCR as diagnose of viral infection. Control plants were inoculated with tungsten for CaLCuV or sap inoculation for TRV and the absence of viral DNA was confirmed by PCR and viral RNA by RT-PCR in these mock-inoculated controls.
Replication	We used three biological replicates of a pool of 10-day-old Col-0 and nik1-1. Differential gene expression methods were optimized to small number of replicates, then we used three biological replicates per treatment. Also the number of three biological replicates of each gene in qRT-PCR has been reported to be sufficient in the analysis of the confidence intervals based on bootstrap. For in vivo experiments (protein-protein interactions, phosphorylation assays, MAPK assay, ROS assay, bacterial growth, etc), we performed at least three biological repeats. Transgene insertion and expression were confirmed by PCR on leaf DNA and qRT-PCR on RNA leaves of transgenic lines. Each biological replicate was constituted by the pooled fraction of RNA from three plants. In addition, we took extra measures to ensure the reproducibility of our results by using two or more independent approaches to address the same question. For example, protein-protein interactions, protein phosphorylation, activation of PTI, activation of NIK1-mediated antiviral signaling.
Randomization	Using completely randomized design, the plants were randomly distributed among the mock and infected groups in the green house. Plants were grown in growth chamber with controlled temperature, photoperiod, humidity and free of insects and any other pathogen. After plant inoculation, they were transplanted to a previously sterilized and mineral-enriched soil. The leaves were visibly examined daily for the complete absence of any other pathogen and uniformly irrigated every two days.
Blinding	Although the concept of "blinded allocation" is more useful for medical researches (patient allocation), this concept can be considered in the study as the randomized allocation of mock and infected groups of plants in the green house.

Reporting for specific materials, systems and methods

We require information from authors about some types of materials, experimental systems and methods used in many studies. Here, indicate whether each material, system or method listed is relevant to your study. If you are not sure if a list item applies to your research, read the appropriate section before selecting a response.

Materials & experimental systems

n/a	Involved in the study	
☐	☒	Antibodies
☒	☐	Eukaryotic cell lines
☒	☐	Palaeontology
☒	☐	Animals and other organisms
☒	☐	Human research participants
☒	☐	Clinical data

Methods

n/a	Involved in the study	
☒	☐	ChIP-seq
☒	☐	Flow cytometry
☒	☐	MRI-based neuroimaging

Antibodies

Antibodies used	Anti-HA-Peroxidase, Roche, Cat # 12013819001, 1:2000; Anti-FLAG M2-Peroxidase, Sigma-Aldrich, Cat # A8592, 1:2000; Anti-GFP, Roche, Cat # 11814460001, 1:1000; Goat anti-Mouse IgG-HRP, Santa Cruz, Cat # sc-2005, 1:10000; Anti-FLAG M2 Affinity gel, Sigma-Aldrich, Cat # 2220; Anti-Phospho-Erk1/2, Cell Signaling, Cat #9101, 1:2000; Goat anti-rabbit IgG-HRP, Santa Cruz, Cat

sc-2004, 1:10000; Anti-AtMPK3, Sigma-Aldrich, Cat# M8318, 1:4000; Anti-AtMPK6, Sigma-Aldrich, Cat# 7104, 1:4000; Anti-Phosphoserine-Peroxidase, Sigma - Aldrich, Cat # SAB5200087, 1:5000; Anti-Phosphotyrosine – Thermo Fisher, Cat # 61-5800, 1:2000. Anti-phosphothreonine - Thermo Fisher, Cat # 71-8200, 1:250
Antibodies were validated by manufacturer and further evaluated using the proper negative controls.

Validation	Antibodies were validated by manufacturer and further evaluated using the proper negative controls.
